# *Salvia miltiorrhiza* ameliorates endometritis in dairy cows by relieving inflammation, energy deficiency and blood stasis

**DOI:** 10.3389/fphar.2024.1349139

**Published:** 2024-04-03

**Authors:** Shiyang Tian, Tianyi Liu, Jingwei Jiang, Xiting Zhao, Yunpeng Fan, Weimin Zhang, Wuren Ma, Tingting Guo, Weiling Wang, Yingqiu Liu

**Affiliations:** ^1^ College of Veterinary Medicine, Northwest A&F University, Yangling, China; ^2^ Institute of Traditional Chinese Veterinary Medicine, Northwest A&F University, Yangling, China; ^3^ Department of Neurosurgery, The First Hospital of Jilin University, Changchun, China; ^4^ Department of Women HealthCare, Changchun Lvyuan Hospital of Traditional Chinese Medicine, Changchun, China; ^5^ College of Chemistry and Pharmacy, Northwest A&F University, Yangling, China

**Keywords:** Salvia miltiorrhiza, traditional Chinese veterinary medicine, blood stasis, inflammation, negative energy balance

## Abstract

**Introduction:** According to traditional Chinese veterinary medicine, endometritis is caused by a combination of Qi deficiency, blood stasis, and external evil invasion. *Salvia miltiorrhiza* is a traditional Chinese medicine that counteracts blood stasis and has additional demonstrated effects in boosting energy and restraining inflammation. *Salvia miltiorrhiza* has been employed in many traditional Chinese prescriptions that have proven effective in healing clinical dairy cow endometritis.

**Methods:** the *in vivo* effect of *Salvia miltiorrhiza* in treating endometritis was evaluated in dairy cows. In addition, bovine endometrial epithelium cell inflammation and rat blood stasis models were employed to demonstrate the crosstalk between energy, blood circulation and inflammation. Network analysis, western blotting, qRT-PCR and ELISA were performed to investigate the molecular mechanism of *Salvia miltiorrhiza* in endometritis treatment.

**Results:** The results demonstrate that treatment with *Salvia miltiorrhiza* relieves uterine inflammation, increases blood ATP concentrations, and prolongs blood clotting times. Four of the six *Salvia miltiorrhiza* main components (SMMCs) (tanshinone IIA, cryptotanshinone, salvianolic acid A and salvianolic acid B) were effective in reversing decreased ATP and increased IL-1β, IL-6, and IL-8 levels in an *in vitro* endometritis model, indicating their abilities to ameliorate the negative energy balance and external evil invasion effects of endometritis. Furthermore, in a blood stasis rat model, inflammatory responses were induced in the absence of external infection; and all six SMMCs inhibited thrombin-induced platelet aggregation. Network analysis of SMMC targets predicted that *Salvia miltiorrhiza* may mediate anti-inflammation via the Toll-like receptor signaling pathway; anti-aggregation via the Platelet activation pathway; and energy balance via the Thermogenesis and AMPK signaling pathways. Multiple molecular targets within these pathways were verified to be inhibited by SMMCs, including P38/ERK-AP1, a key molecular signal that may mediate the crosstalk between inflammation, energy deficiency and blood stasis.

**Conclusion:** These results provide mechanistic understanding of the therapeutic effect of *Salvia miltiorrhiza* for endometritis achieved through Qi deficiency, blood stasis, and external evil invasion.

## 1 Introduction

Endometritis is a high incidence disease in dairy cows that occurs *postpartum*. Its symptoms can vary depending on the disease etiology but typically include changes in the color, consistency, and amount of vaginal discharge. Endometritis has serious consequences for dairy herds, including reproductive disfunction, extended calving intervals, secondary diseases, and even increased elimination rates (McKay et al., 2023).

In modern veterinary medicine, endometritis is typically treated with antibiotics (Taylor et al., 2019; Cicinelli et al., 2021). While bacterial infection is a hallmark symptom, physical trauma and chemical irritation have also been associated with endometriosis (Potter et al., 2010). Furthermore, the overuse of antibiotics has led to drug resistance, immunity decline, and problems associated with drug metabolism (Woodd et al., 2019). Consequently, alternative therapies have become increasingly important (Scoggin, 2016).

According to traditional Chinese veterinary medicine (TCVM), endometritis is caused by a combination of Qi deficiency, blood stasis, and external evil invasion (Zhang et al., 2021). When giving birth, cows naturally lose much of their Qi, often translated as “vital energy” (Wang et al., 2017; Bao, 2020). Milk production and *postpartum* recovery require additional Qi and contribute to a condition of *postpartum* negative energy balance (NEB). Excessive NEB may lead to metabolic disorders and impaired fertility (Churakov et al., 2021). NEB reduces the cow’s immunity and causes lipolysis, ketogenesis and endocrinological changes, which can lead to more severe endometritis, especially in high yielding cows (Cheong et al., 2011; Krnjaić et al., 2022). The NEB can be determined using plasma thresholds of β-hydroxybutyrate, non-esterified fatty acids and glucose (Macrae et al., 2019; Churakov et al., 2021). Additionally, blood stasis, defined by sub-optimal blood flow rather than by thrombosis (Huang et al., 2021), happens broadly during female pregnancy and recovers spontaneously a few days after delivery. However, insufficient Qi to provide power for motion may result in continued *postpartum* blood stasis (Li et al., 2014). Because infection is the primary pathogenic mechanism of endometritis (Sheldon et al., 2020), exogenous pathogens and the toxins that they secrete are direct factors in inducing the inflammation that underlies endometriosis, as well as the source of “external evil” in TCVM (Pascottini and LeBlanc, 2020; Zhang et al., 2021). Therefore, the principles of TCVM provide a complementary perspective toward understanding endometritis.

Many traditional Chinese prescriptions have proven effective in healing clinical dairy cow endometritis, including *Salvia miltiorrhiza* Bunge *(Lamiaceae; Salviae miltorrhizae radix et rhizoma*) (Arlt et al., 2009). Traditionally, *Salvia miltiorrhiza* belongs to a class of drugs that invigorate blood circulation and eliminate stasis (Zhao et al., 2022). Thus, many studies have focused on its activity and mechanism in blood circulation; though recent studies have addressed the effects of *Salvia miltiorrhiza* in regulating energy and anti-inflammation (Wang et al., 2022; Ma et al., 2023; Zou et al., 2023). Additionally, a traditional Chinese medicine formulation investigated by our research team (comprising *Salvia miltiorrhiza* and other herbal medicines), named Danlianhua Uterine infusion (DLH), demonstrated therapeutic efficacy across various forms of endometritis (Li et al., 2019). The purpose of our study was to evaluate whether the therapeutic effect of *Salvia Miltiorrhiza* in DLH is related to negative energy balance, blood stasis and anti-inflammation, and to explore its molecular mechanism in the treatment of endometritis. To clarify the molecular mechanism of *Salvia miltiorrhiza* in tonifying Qi, promoting blood circulation, and inhibiting inflammation, we sought to investigate its molecular mechanisms in endometritis treatment, guided by TCVM theory.

## 2 Materials and methods

### 2.1 Animals and ethics statement

Dairy cows were from a farm in Ningxia province of China. Holstein cows aged 22–44 months (1–2 parity), 21–35 days *postpartum* were included in the study. SD rats 6–7 weeks of age, purchased from Chengdu Dashuo experimental animal Co., LTD (Xianyang, Shaanxi, China), were acclimated for 1 week before experiments. All procedures for animal experiments were approved by the Animal Ethical and Welfare Committee of Northwest A&F University (Approval No. 2021012).

### 2.2 *Salvia miltiorrhiza* extracts preparation

*Salvia miltiorrhiza* was purchased from Beijing Tongrentang Co., LTD (batch number: 2012027. Date of production: 25 December 2020). *Salvia miltiorrhiza* was extracted with 70% ethanol at a ratio of 1:10 (solid: liquid) at 50°C for 1 h, for a total of three repeats. All extracts were combined, filtered, precipitated, and then condensed to one-tenth of the original volume using a rotary evaporator. The resultant suspension was packaged, sealed, and autoclaved.

### 2.3 Qualitative and quantitative analysis of *Salvia miltiorrhiza* extracts

The main compounds in the *Salia miltiorrhiza* extracts, tanshinones and salvianolic acids, were determined by TLC, UPLC and HPLC. The standards tanshinone I (TI) tanshinone IIA (TIIA), dihydrotanshinone I (DI) and cryptotanshinone (CT) were used for analysis of the tanshinones, while salvianolic acid A (SAA) and salvianolic acid B (SAB) were used as the standards for salvianolic acids.

TLC analysis was performed with GF254 silica gel plate. The developing solvents for tanshinones and salvianolic acids were petroleum ether: ethyl acetate: cyclohexane (4:3:2) and dichloromethane: trichloromethane: ethyl acetate: methanol: formic acid (4:6:8:1:4), respectively. The tanshinones and salvianoic acids were observed by an ultraviolet lamp under 254 nm.

UPLC analysis was performed by UPLC system (LC-30A03030623), with the following system parameters: column temperature 30°C; injection volume 10 μL; flow rate 0.3 mL/min; detection wavelength 254 nm. The mobile phase was acetonitrile (A) − 0.01% formic acid in water (B). The gradient program was as follows: 90%–40% B for 0–5 min; 40%–20% B for 5–10 min; 20% B for 10–14 min; 20%–10% B for 14–17 min.

For HPLC analysis, an Acclaim C18 column (5 μm, 4.6*250 mm) was used, and the mobile phase was acetonitrile (A) − 0.01% formic acid in water (B). The parameters were: column temperature 35°C; injection volume 10 μL; flow rate 1.0 mL/min; and detection wavelength 254 nm. The gradient program of tanshinones was as follows: 90% B for 0–5 min; 90%–60% B for 5–20 min; 60%–40% B for 20–30 min; 40%–33% B for 30–50 min. The analysis system for salvianolic acids was: 83%–77% B for 0–15 min; 77%–75% B for 15–30 min; 75%–65% B for 30–40 min.

### 2.4 Dairy cow diagnosis

Due to the lack of a diagnostic gold standard, endometritis in dairy cows is a controversial issue (Kasimanickam et al., 2005). Most clinical diagnosis of endometritis is determined by uterine mucus and polymorphonuclear neutrophil (PMN) content. Therefore, in this study, *postpartum* healthy and endometritis dairy cows were diagnosed both by vaginal discharge score (VDS) and %PMN. VDS was evaluated according to uterine mucus as follows: 0, complete transparent or translucent mucus; 1, mucus contain white or grayish-white purulent spots; 2, mucus contain less than 50% pus; 3, mucus contain over 50% pus (Diaz-Lundahl et al., 2021). For PMN evaluation, a sterile cytology brush was applied against the uterine body and rotated 3 times and then was gently rolled onto a clean microscope slide to prepare an endometrial cytology slide. At 400 × magnification, a total of 300 representative epithelial cells and PMN were counted in several fields, and the percentage of PMNs was calculated. Cows with VDS≥2 and %PMN >5 were defined as having endometritis (Wagener et al., 2017), and healthy cows were certified by VDS = 0, %PMN <5, and no signs of puerperal disease (either uterine or extrauterine). None of the cows were undergoing antibiotic or anti-inflammatory therapy at the time of experimentation.

### 2.5 Dairy cow experiments

Ten healthy cows and twenty cows with endometritis (randomly divided into the endometritis and SM groups; *n* = 10 per group) were included in the study. The cows within group SM received 100 mL of *Salvia miltiorrhiza* extract injected into the uterine cavity, administered once every other day for three consecutive days, while cows in the healthy and endometritis groups were perfused with 100 mL of saline. All the experimental cows were individually housed in barn stalls with sand as the bedding material for 24 h. The cows were withheld from any drug treatment and were allowed to feed freely with the same forage (corn silage; alfalfa hay; whole cotton seed; beet granules; corn flakes; corn meal; soybean meal; extruded soybean; corn gluten powder; rapeseed meal; corn distilled soluble grain; calcium fatty acid; sodium bicarbonate; urea). All animals were perfused once every other day for three consecutive days. Twelve hours after the last perfusion, samples were collected for evaluation of VDS, %PMN, coagulation and ATP concentration. ATP contents in whole blood were detected with an ATP assay kit (Beyotime Biotechnology, Shanghai, China). Whole blood was lysed using the lysis buffer provided in the kit. After lysis, the samples were centrifuged at 12,000 g for 10 min at 4°C. The supernatant was collected for protein concentration measurement, and the ATP contents in 100 μg protein/sample were subjected to ATP measurement. The chemiluminescence intensity was detected using a Multiscan microplate spectrophotometer (Molecular Devices, Sunnyvale, CA, United States). The prothrombin time (PT), active part thrombin time (APTT), thrombin time (TT) and fibrinogen (FIB) were measured using plasma obtained from anti-coagulated blood (sodium citrate: blood = 1:9; 800 g, 10 min) using a Coagulator system (MD PACIFIC, Tianjin, China).

### 2.6 Cell culture, cell viability and ATP content determination

Bovine endometrial epithelium cells (BEECs), purchased from BeNa Culture Collection (Beijing, China), were cultured in DMEM medium containing 10% newborn bovine serum (37°C; 5% CO_2_) and treated with the required drugs for the indicated times as needed for individual experiments. After drug treatment, cell viability was determined using WST-1 assay kits (Beyotime Biotechnology, Shanghai, China), with the OD_450nm_ detected by a Multiscan microplate spectrophotometer (Molecular Devices, Sunnyvale, CA, United States). ATP contents in BEECs were detected as described in 2.5.

### 2.7 Blood stasis rat model studies

SD rats were randomly grouped into the blood stasis or control group (*n* = 10 per group). Rats in the blood stasis group were subcutaneously injected with 0.8 mg/kg epinephrine twice, with 4 h interval. Rats in the control group were injected with the same volume of saline. Twenty-four hours after the last injection, the rats were anesthetized with ether, and blood was drawn from the abdominal aorta.

For whole blood viscosity detection, whole blood was anti-coagulated with heparin sodium (heparin sodium: blood = 1:9). Values with shear rate of 1, 3, 10, 30, 50, and 200 times/s were detected using an Automatic blood viscosity dynamic analyzer (Nanfang Numerical Control, Chongqing, China).

For assessment of coagulation indices (PT, APTT, TT, and FIB), blood was anti-coagulated with sodium citrate (sodium citrate: blood = 1:9) and centrifuged (800 g, 10 min). After centrifugation, the upper layer plasma was incubated with TIIA, CT, DI, SAA,SAB, TI or DMSO for 5 min, and the coagulation indices were detected with a Coagulator (MD PACIFIC, Tianjin, China).

Uterine tissues were freshly preserved in 4% paraformaldehyde for 48 h, followed by dehydration, clearing, embedding, and thin-sectioning of tissues. The sections were dewaxed in xylene, re-hydrated in gradient ethanol (100%–70%), and immersed in hematoxylin for 4 min and then eosin for 1 min. Subsequently, slices were dehydrated, cleared, mounted with neutral balsam mounting medium, and observed by microscopy (EX31, SOPTOP, China, Ningbo).

### 2.8 Platelet aggregation measurement

Platelet aggregation experiments were performed in accordance with our previous study (Liu et al., 2018). To prepare washed platelets (WPs), blood of dairy cows was anti-coagulated with acid-citrate-dextrose (85 mM trisodium citrate, 66.6 mM citrate and 111 mM glucose; acid-citrate-dextrose: blood = 1:6). After centrifugation (150 g, 10 min), the platelet precipitate was washed once with washing buffer (138 mM NaCl, 2.8 mM KCl, 0.8 mM MgCl_2_, 0.8 mM NaH_2_PO_4_, 10 mM HEPES, and 5 mM EDTA, pH 7.4) and further centrifuged (250 g, 10 min). WPs were obtained by suspending the platelet pellet in suspension buffer (138 mM NaCl, 2.8 mM KCl, 0.8 mM MgCl_2_, 0.8 mM NaH_2_PO_4_, 10 mM HEPES, 5.6 mM dextrose, and 1 mM CaCl_2_, pH 7.4) and adjusting to 2 × 10^8^ platelets/mL. Platelet aggregation experiments were performed using a four channel aggregometer (TECHLINK BIOMEDICAL, Beijing, China).

### 2.9 ELISA assay of cell protein contents

The concentrations of an array of proteins in BEECs, WPs or uterine tissues were evaluated by ELISA. After drug treatment, cells or tissues were lysed and centrifuged (4°C, 12,000 g, 5 min), and the supernatant was collected for analysis of secreted IL-1β, IL-6, and IL-8 levels. For detection of ARHGEF12, RhoA, ROCK, AKT, P38 and ERK, the pellets were lysed and centrifuged (4°C, 12,000 g, 5 min) to obtain cytoplasm. The protein contents in uterine tissues were detected using tissue lysates. All ELISA kits were purchased from FANKEW (Biotechnology, Shanghai, China), and all procedures were performed according to the ELISA kit instructions.

### 2.10 Western blot analysis

BEEC or WP lysate was quantified with a BCA protein assay kit (Solarbio, Beijing, China), and equal amounts of protein were subjected to SDS-polyacrylamide gel electrophoresis. After transfer to a polyvinylidene difluoride membrane, the proteins were detected with primary antibodies (phospho-p38 Thr180/Tyr182 polyclonal antibody, p38 polyclonal antibody, Phospho-Erk1/2 Thr202/Tyr204 monoclonal antibody, Erk1/2 monoclonal antibody, SRC monoclonal antibody, αIIbβ3 monoclonal antibody, phosphor-eNOS ser1177 polyclonal antibody, eNOS monoclonal antibody, or tubulin polyclonal antibody). Antibodies for P-P38, P38, P-ERK, ERK and tubulin were from Cell Signaling Technology; antibodies for SRC and αIIbβ3 were from HUABIO; P-eNOS antibody was from Affinity Biosciences; and eNOS antibody was from Abcam. HRP conjugated secondary antibodies (goat anti-rabbit and goat anti-mouse IgG antibodies were from Solarbio Science & Technology), and Immobilon Western detection reagents were as described previously (Liu et al., 2016). Chemiluminescence images were obtained and analyzed with a chemiluminescence gel imaging system (P&G Science & Technology, Shanghai, China).

### 2.11 Quantitative real-time PCR (qRT–PCR)

Total RNA was isolated from BEECs using TRIzol reagent (Thermo Fisher Scientific, MA, United States). Gene expression was then measured by qRT-PCR using the GoTaq 1-Step RT-qPCR System (Promega, WI, United States) on a CFX96 Real-Time PCR Detection System (Bio-Rad, CA, United States). The verified mRNA primers are listed in Supplementary Table S1. The cycling conditions were as follows: Pre-denaturation at 95°C for 10 min; followed by 40 cycles of denaturation at 95°C for 10 s, annealing at 60°C for 30 s and extension at 72°C for 30 s. The relative expression levels of target genes and differentially expressed miRNAs were normalized to 36b4 expression for each respective sample and were calculated by the 2-ΔΔCq method (CFX Manager Software, version 1.6, Bio-Rad).

### 2.12 Network pharmacology analysis

Predicted targets of the *Salvia miltiorrhiza* main components (SMMCs) were searched in ETCM (http://www.tcmip.cn/ETCM/index.php/Home/Index/) and TCMSP (http://tcmspnw.com) databases and uploaded to STRING. The targets that matched topological parameters of betweenness >420.478921 and closeness >0.000910 were evaluated by KEGG enrichment analysis, and a network diagram of the correlation between SMMCs and target genes was drawn using Cytoscape version 3.5.1.

### 2.13 Statistical analysis

The mean and standard error were calculated for all experimental groups. The data were subjected to one-way analysis of variance followed by Dunnett’s test to determine significant differences. Statistical analysis was performed using SigmaStat software ver. 3.5 (Systat Software, San Jose, CA, United States). *p*-value < 0.05 was considered as significant.

## 3 Results

### 3.1 *Salvia miltiorrhiza* relieves endometritis in dairy cows

**FIGURE 1:**
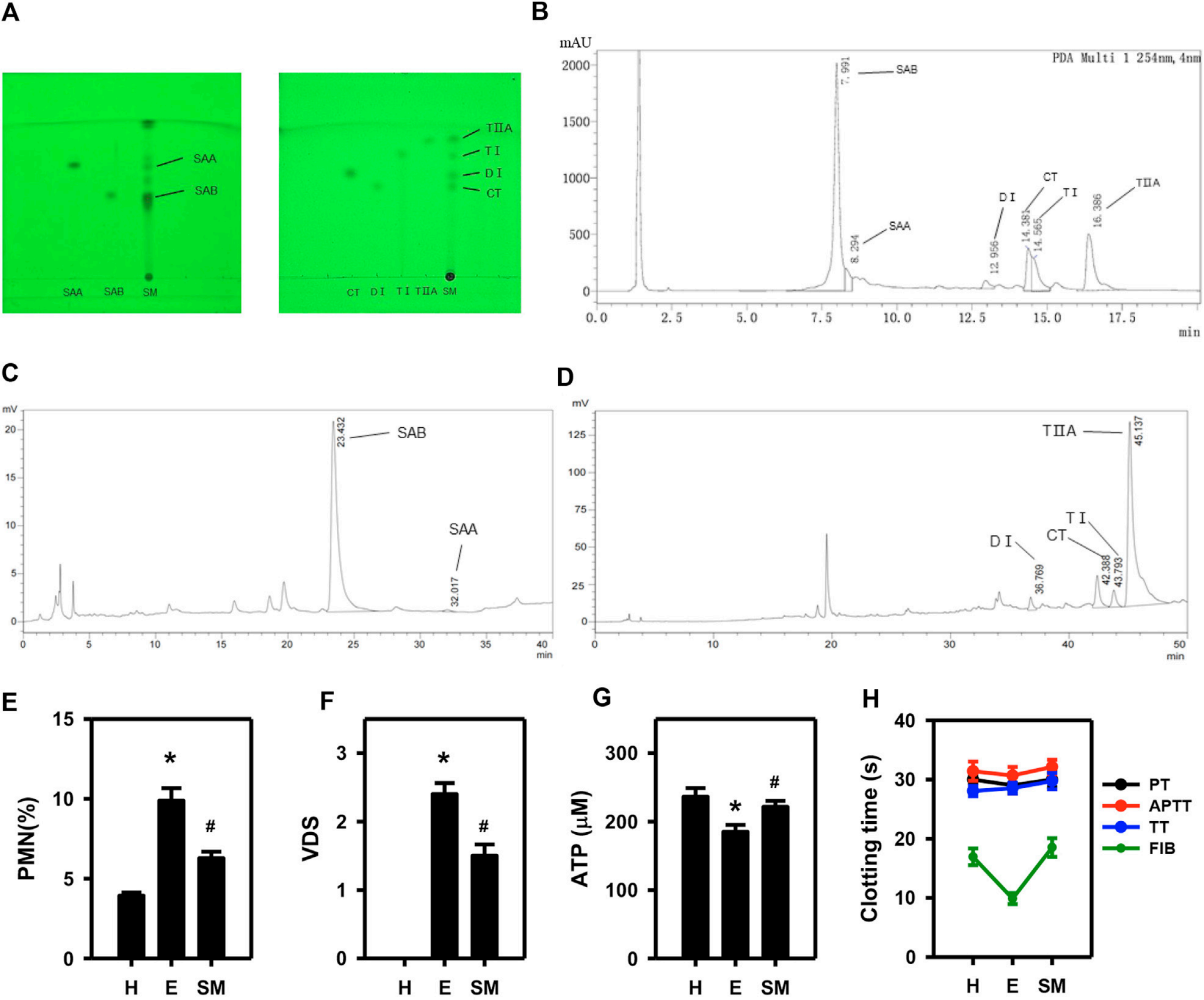
Examination of the active constituents of *Salvia miltiorrhiza* and their role in mitigating endometrial inflammation in dairy cows. Analysis of extracts of *salvia miltiorrhiza* by TLC **(A)**, UPLC **(B)** and HPLC **(C, D)**. The effect of *Salvia miltiorrhiza* in endometritis dairy cows was evaluated via assessment of the %PMN **(E)** and VDS **(F)** in exudate from uterine swabs, blood ATP concentration **(G)**, and four coagulation indices **(H)**. H, E and SM stands for healthy, endometritis and *Salvia miltiorrhiza*-treated endometritis groups, respectively. PT, APTT, TT, and FIB indicates prothrombin time, active part thrombin time, thrombin time and fibrinogen of four coagulation indices, respectively. (*n* = 10; ^*^ *p* < 0.05 vs. H; ^#^ *p* < 0.05 vs. **(E)**.

Qualitative and quantitative analyses of *Salvia miltiorrhiza* extracts were performed using TLC, UPLC and HPLC. The *Salvia miltiorrhiza* extracts contained SAB, SAA, TI, CT, TIIA, and DI (Figures 1A–D, Supplementary Table S2). To evaluate the *in vivo* efficacy of *Salvia miltiorrhiza* in treating endometritis, we assessed the VDS and %PMN of dairy cows. Cows that met the criteria for endometritis (VDS >2 and %PMN >5) (Wagener et al., 2017) were treated with *Salvia miltiorrhiza* or saline (control) for 3 consecutive days and then were re-evaluated 12 h after the last perfusion. The results demonstrate that endometritis cows perfused with *Salvia miltiorrhiza* extract as compared to saline (E) had a statistically reduced %PMN and a trend towards lower VDS, though the treatment was not sufficient to cure endometritis completely to the level of the healthy cows (H) (Figures 1E,F; Supplementary Table S3). Moreover, *Salvia miltiorrhiza* was effective in up-regulating blood ATP content and prolonging the plasma FIB clotting time (Figures 1G,H). These results indicate that a 3-day *Salvia miltiorrhiza* treatment may partially alleviate endometritis in dairy cows.

### 3.2 *Salvia miltiorrhiza* main components (SMMCs) relieve inflammatory reactions in cultured bovine endometrial epithelium cells (BEECs)

**FIGURE 2:**
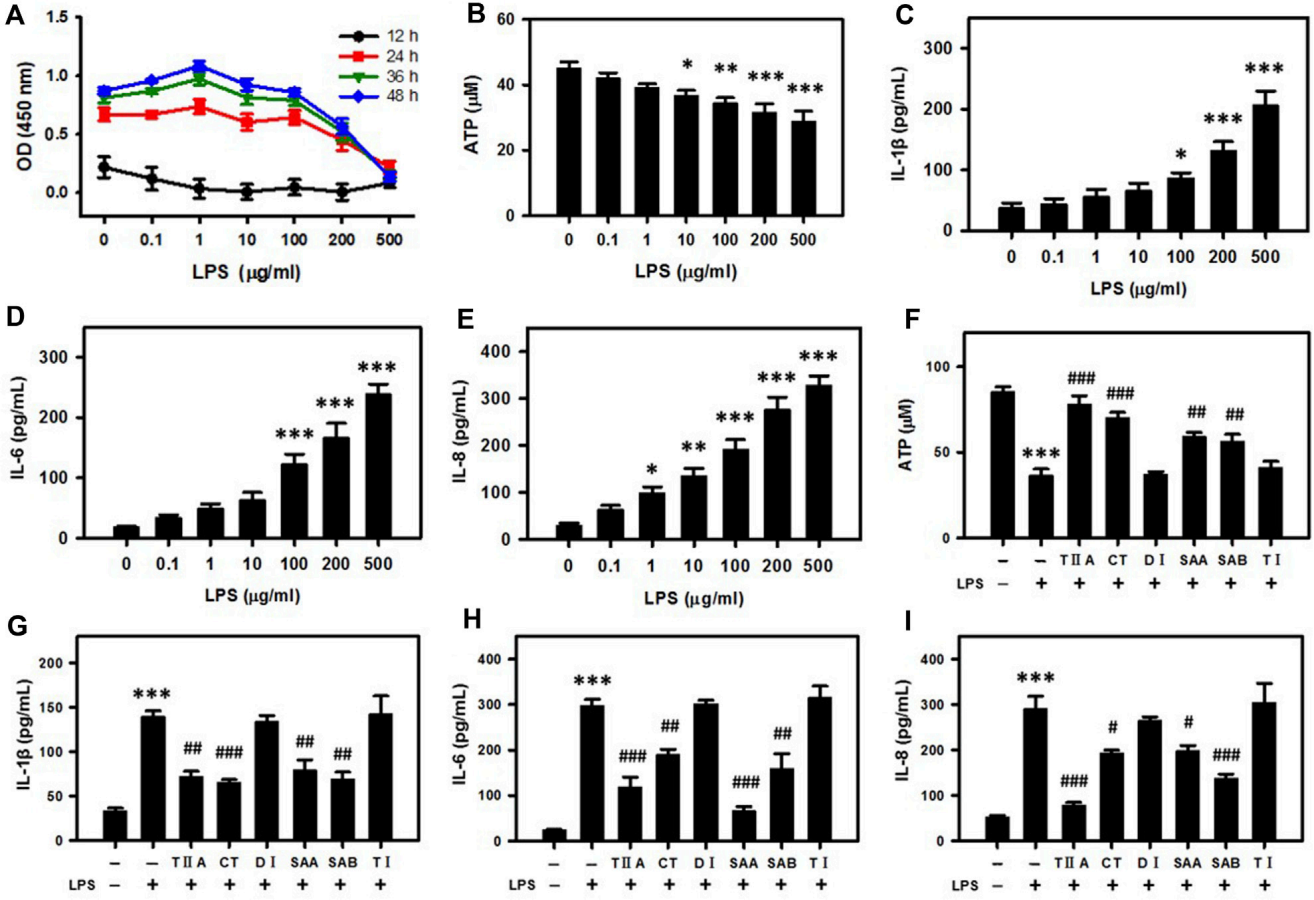
SMMCs attenuate energy loss and inflammatory responses in bovine endometrial epithelium cells (BEECs). Viability assay of BEECs after exposure to increasing doses of LPS. The cells were evaluated at different times coded by color **(A)**. Concentration dependent effect of LPS on the cellular ATP content **(B)**. IL-1β, IL-6 and IL-8 secretion levels after exposure to increasing doses of LPS **(C–E)**. Effect of SMMCs in preventing LPS-stimulated ATP decrease **(F)**. Effect of SMMCs in preventing LPS-stimulated IL-1β, IL-6 and IL-8 secretion **(G–I)**. Values represent means ± SE (^*^ *p* < 0.05, ^**^ *p* < 0.01, ^***^ *p* < 0.001 vs. control group; ^#^ *p* < 0.05; ^##^ *p* < 0.01; ^###^ *p* < 0.001 vs. LPS group). *n* = 3 for A, F-I; *n* = 5 for B; *n* = 6 for C-E.

To study the molecular mechanism of *Salvia miltiorrhiza* in endometritis treatment, we selected BEECs as an *in vitro* model for endometritis due to their important role in preventing or promoting inflammation development in the uterus (Shen et al., 2022). An inflammation reaction was triggered in the BEECs by applying increasing doses of lipopolysaccharide (LPS) over a time course. According to the results, cell proliferation was inhibited in a dose-dependent manner after 24–48 h LPS treatment (Figure 2A). Because prolongation of the incubation time past 24 h did not substantially influence the tendency, we selected 24 h incubation for subsequent experiments. Incubation of BEECs with LPS suppressed the cellular ATP content in a dose-dependent manner (Figure 2B), while the secreted levels of pro-inflammatory cytokines IL-1β, IL-6, and IL-8 were simultaneously upregulated (Figures 2C–E), thus verifying the establishment of the model.

To evaluate molecular components responsible for *Salvia miltiorrhiza* therapeutic activity, we repeated the viability assays after application of six SMMCs in conjunction with LPS. TIIA, CT, SAA and SAB reversed the LPS-dependent decrease in ATP levels, while DI and TI failed to block this decrease (Figure 2F). In addition, LPS-stimulated IL-1β, IL-6, and IL-8 secretion levels were significantly inhibited by TIIA, CT, SAA, and SAB, but not by DI and TI (Figures 2G–I). These results indicate that LPS triggers cellular energy loss and inflammation, and that TIIA, CT, SAA and SAB can reverse the external evil invasion modeled by LPS exposure.

### 3.3 SMMCs relieve the negative energy balance in BEECs

**FIGURE 3:**
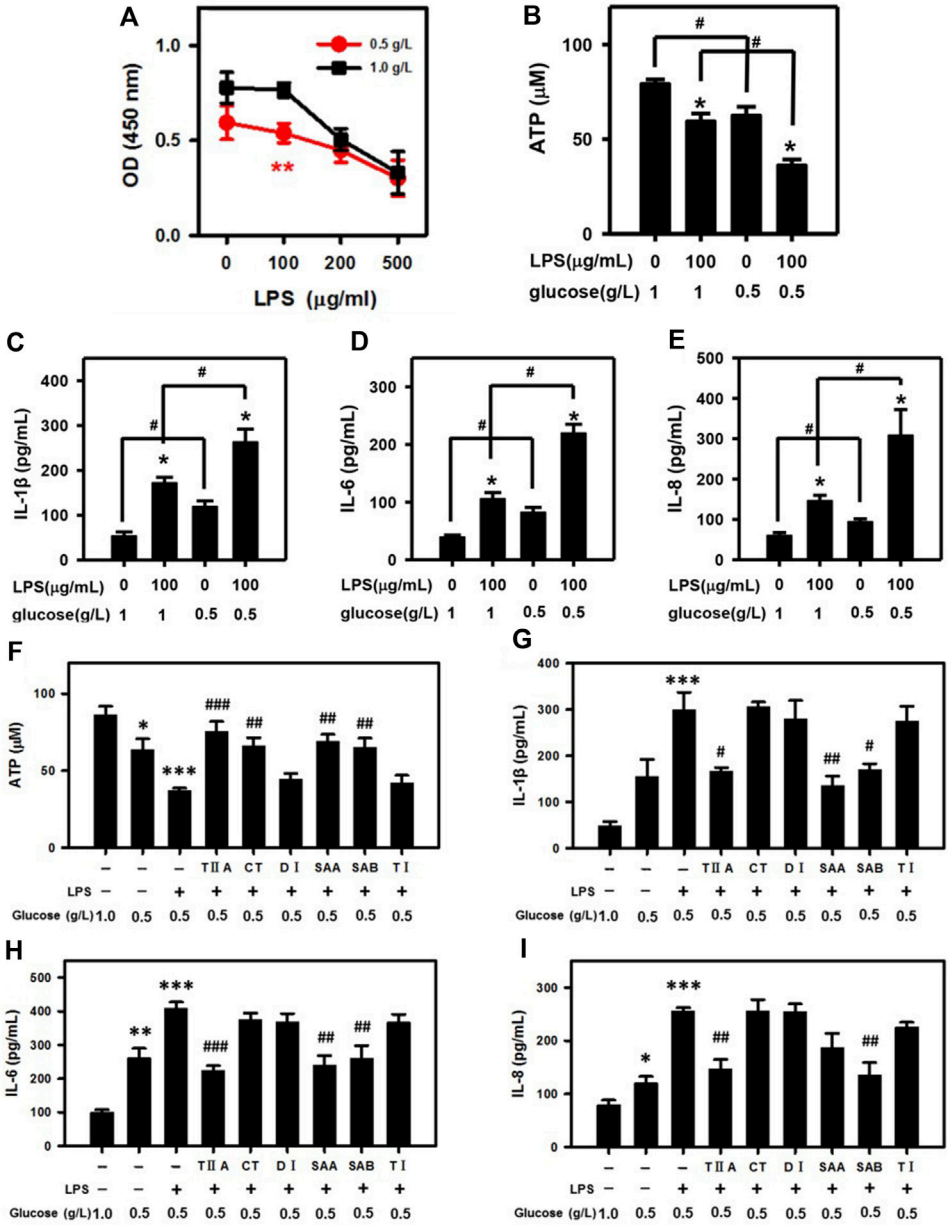
SMMCs protect NEB cells from inflammatory responses. Viability assay of BEECs cultured for 24 h in normal DMEM (1.0 g/L) or low glucose DMEM (0.5 g/L; NEB conditions) with increasing amounts of LPS **(A)**. Effect of low glucose in exacerbating LPS-induced ATP deficiency **(B)**. Effect of low glucose on LPS-stimulated IL-1β **(C)**, IL-6 **(D)** and IL-8 **(E)** secretion. Effect of SMMCs in preventing LPS- and low glucose-triggered ATP decrease **(F)**. Effect of SMMCs in inhibiting LPS-stimulated IL-1β **(G)**, IL-6 **(H)** and IL-8 **(I)** secretion under low glucose conditions. Values represent means ± SE. (A: ^**^ *p* < 0.01 vs. 100 μg/mL LPS and 1.0 g/L glucose; B–E: ^*^ *p* < 0.05 vs. same glucose concentration group, ^#^ *p* < 0.05 vs. same LPS concentration group; F-I, ^*^ *p* < 0.05, ^**^ *p* < 0.01, ^***^ *p* < 0.001 vs. control group, ^#^ *p* < 0.05; ^##^ *p* < 0.01; ^###^ *p* < 0.001 vs. LPS+ 0.5 g/L glucose group). *n* = 6 for A; *n* = 3 for B-I.

Because glucose is the main source of cell energy, we mimicked conditions of NEB by culturing BEECs in low glucose DMEM (0.5 g/L; compared 1 g/L in standard DMEM). According to our cell viability assays, 100 μg/mL LPS selectively suppressed NEB cell proliferation in low glucose BEECs (Figure 3A). Furthermore, the ATP level was reduced in low glucose media vs. standard media and was most dramatically reduced in BEECs with a combination of both low glucose and LPS treatment (Figure 3B). Low glucose also led to increased IL-1β, IL-6, and IL-8 secretion levels that were most highly increased upon co-treatment with LPS (Figures 3C–E). Notably, the effects of low glucose media in enhancing the LPS-mediated suppression of ATP and increase in IL-1β, IL-6, and IL-8 levels were reversed by TIIA, CT, SAA and SAB (Figures 3F–I). These results suggest that SMMCs can restore NEB effects associated with endometritis.

### 3.4 Inflammatory cytokine secretion is activated in a rat blood stasis model, and SMMCs inhibit platelet aggregation

**FIGURE 4:**
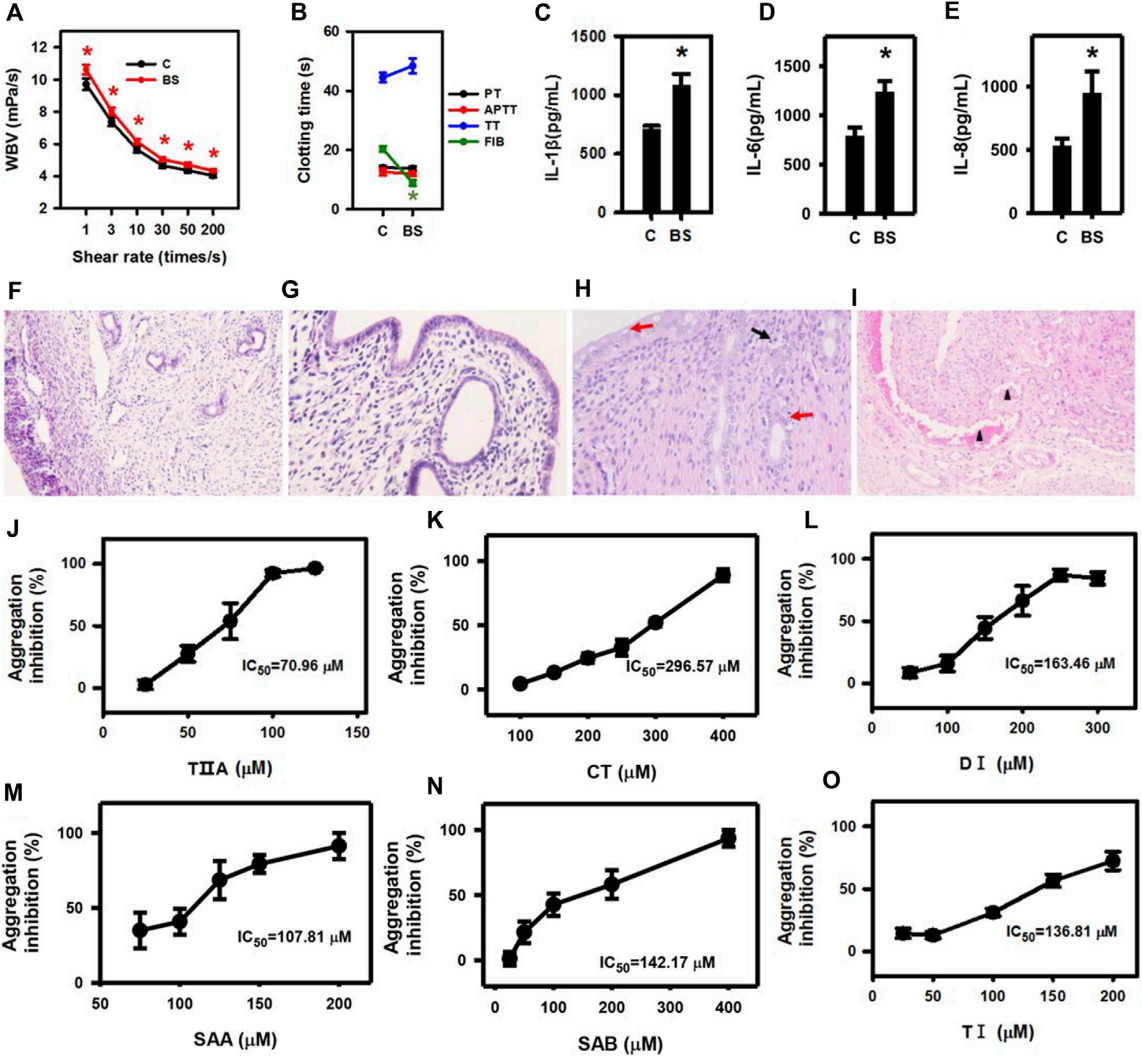
Inflammatory cytokine secretion is triggered in a rat blood stasis model, and SMMCs inhibit platelet aggregation. Whole blood viscosity **(A)** and four coagulation indices **(B)** of rats in control and blood stasis groups. IL-1β **(C)**, IL-6 **(D)** and IL-8 **(E)** concentrations in uterine lysates. **(C)** control; BS: blood stasis. The normal structure of the uterus in the control group **(F)** 100×, **(G)** 400×. In the blood stasis group, marked blood stasis in the myometrium (triangle) was observed at lower magnification (×100) of blood stasis rat uteri **(H)**. The mucosal epithelium of the uterus (400×) showed vacuolar degeneration (red arrow) and scattered necrosis (red arrow) with mild neutrophil infiltration (black arrow) **(I)**. Anti-platelet effect and IC_50_ of TIIA, CT, DI, SAA, SAB and TI are shown in **(J–O)**. Values represent means ± SE (^*^ *p* < 0.05; *n* = 10 for **(A,B)**; *n* = 3 for **(C–E)**; *n* = 5 for **(J–O)**.

To evaluate the role of SMMCs in relieving blood stasis caused by endometritis, we employed a rat blood stasis model. After adrenaline injection, the whole blood viscosity was significantly higher and the plasma FIB content was significantly decreased in the blood stasis group as compared to the control group (Figures 4A,B), thus confirming the establishment of the rat blood stasis model. Furthermore, IL-1β, IL-6, and IL-8 concentrations in the uteruses of blood stasis rats compared with control rats were significantly upregulated (Figures 4C–E). In the uteri of blood stasis rats, vacuolar degeneration and scattered necrosis of mucosal epithelium with mild neutrophil infiltration were observed, and the myometrium showed marked blood stasis (Figures 4F–I). Notably, these results indicate that inflammatory responses may occur in conjunction with blood stasis and in the absence of external infection.

Next, we added the SMMCs to the blood coagulation assay. Platelet aggregation stimulated by thrombin was inhibited by all the six SMMCs, with varying IC_50_ (Figures 4J–O), though up to 1 mM SMMCs failed to influence plasma coagulation (data not shown). These results are consistent with the possibility that SMMCs may relieve blood stasis by directly regulating the function of platelets.

### 3.5 Network pharmacology analysis to predict SMMC targets

**FIGURE 5:**
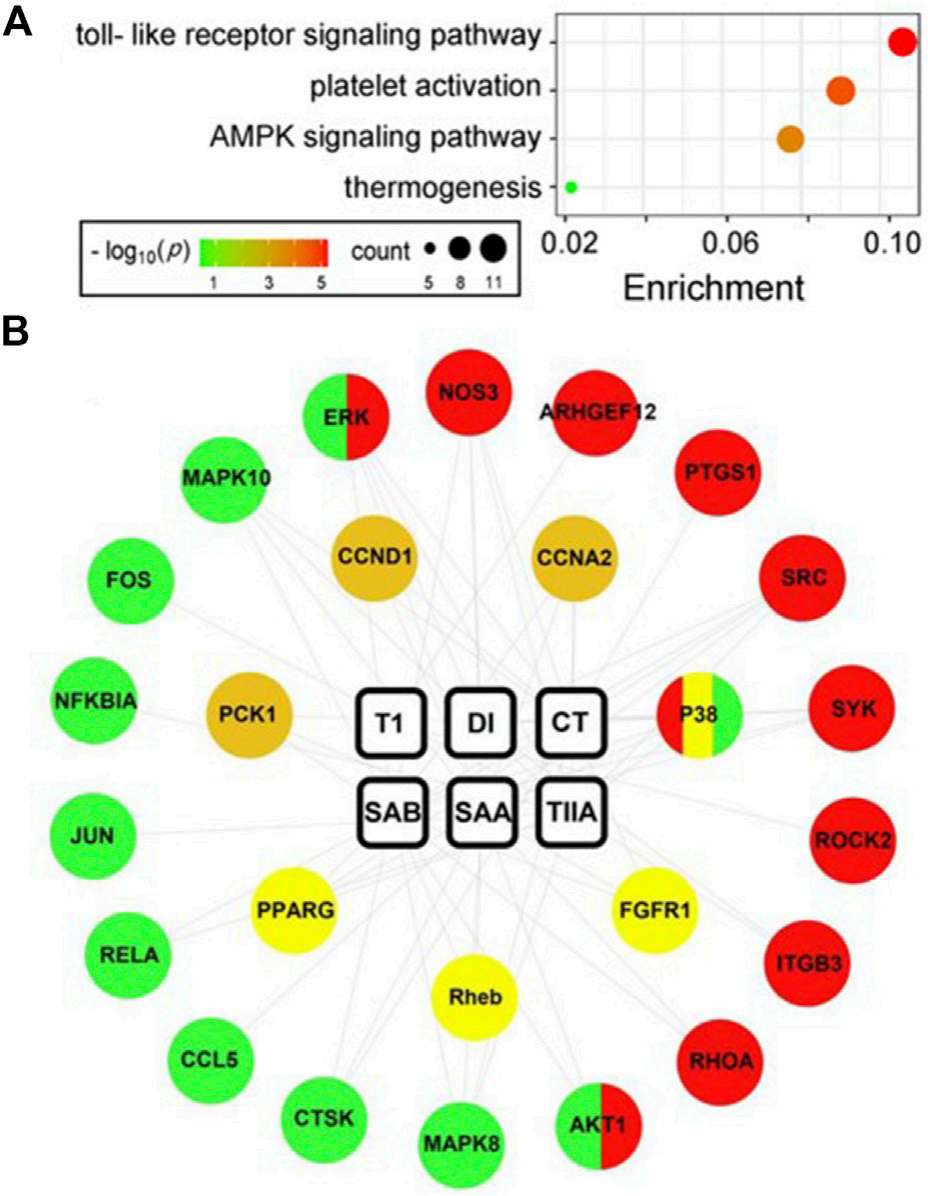
Network pharmacology analysis of SMMCs targets. SMMC targets (*n* = 89) were predicted using the TCMSP and ETCM databases (see Supplementary Table S5). KEGG enrichment analysis of the 89 predicted SMMCs targets is shown in the bubble chart. The color and size of the bubbles reflects–log_10_(*p*) and target count, respectively **(A)**. The network chart indicated the correlation between SMMCs and the targets enriched in the four main pathways: the Toll-like receptor signaling (green), Platelet activation (red), AMPK signaling (tawny) and Thermogenesis (yellow) pathways. The correlations between SMMCs and targets are indicated by gray lines **(B)**.

To explore the molecular mechanisms of the six SMMCs in ameliorating endometritis, we evaluated potential pharmacological targets using TCMSP (http://tcmspnw.com) and ETCM (http://www.tcmip.cn/ETCM/index.php/Home/Index/) databases. The predicted molecular targets for SMMCs (483 in total) are listed in Supplementary Table S4. After uploading the targets to STRING, 89 passed our screening criteria of Betweenness>420.48, Closeness>0.00091, and Degree>20 (Supplementary Table S5). To evaluate the functions of these 89 potential targets, we performed functional annotation according to the KEGG database, which demonstrated enrichment in 4 main pathways: the Toll-like receptor signaling pathway (which correlates with inflammation), the Platelet activation pathway (which correlates with blood stasis), and the AMPK signaling and Thermogenesis pathways (which correlate with energy) (Figure 5A). The predicted molecular targets in these 4 pathways and their correlations with the 6 SMMCs are presented in a network graph (Figure 5B), and the corresponding protein names of the predicted target genes are presented in Supplementary Figures S1–S4 and listed in Supplementary Table S6. These results provide mechanistic understanding at the molecular level of the ability of SMMCs to target processes associated with inflammation, blood stasis, and energy.

### 3.6 Evaluation of molecular targets associated with inflammation

**FIGURE 6:**
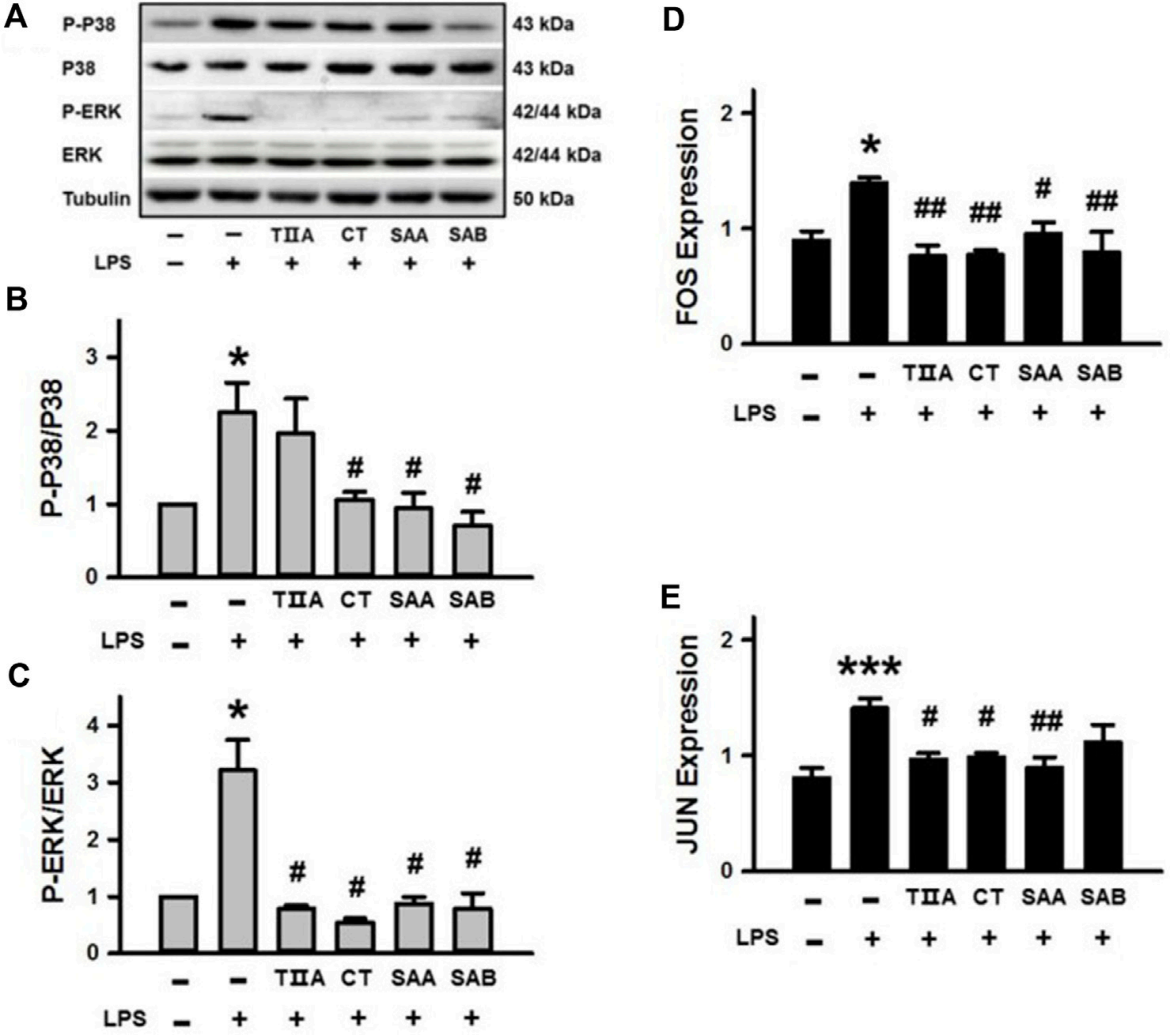
Verification of the effects of SMMCs on Toll-like receptor signaling pathway targets. Western blotting assay of BEECs cultured for 24 h with or without 100 μg/mL LPS and four SMMCs. The protein phosphorylation of P-P38/P38 and P-ERK/ERK were evaluated, and tubulin was also tested as a loading control **(A)**. Quantification of the relevant ratios of P-P38/P38 **(B)** and P-ERK/ERK **(C)**. Gene expression of FOS and JUN were determined by qRT-PCR **(D, E)**. Values represent means ± SE (^*^ *p* < 0.05, ^***^ *p* < 0.001 vs. control group; ^#^ *p* < 0.05, ^##^ *p* < 0.01 vs. LPS group; *n* = 3 for B-C; *n* = 4 for **(D, E)**.

To confirm the molecular function of the four SMMCs (TIIA, CT, SAA and SAB) that were demonstrated to relieve inflammatory responses (Figures 2F–I) and were predicted to target the Toll-like receptor signaling pathway (Supplementary Figure S1), we performed Western blot analyses of LPS-stimulated BEECs. The results demonstrate that LPS triggers phosphorylation of P38 and ERK, and that LPS-triggered phosphorylation is suppressed by co-treatment with CT, SAA and SAB for p38 and all four SMMCs for ERK (Figures 6A–C). Furthermore, all four SMMCs reversed the LPS-induced elevation in FOS mRNA expression, while TIIA, CT and SAA reversed the LPS-induced elevation in JUN mRNA expression (Figures 6D,E). However, the effect of the SMMCs on the expression and/or phosphorylation of AKT1, RELA, NFKBIA, MAPK8, MAPK10, CTSK, and CCL5 could not be confirmed (data not shown). Collectively, the above results suggest that SMMCs can prevent LPS-triggered inflammation through inhibition of P38, ERK and AP-1 (FOS and JUN), which might explain the functions of *Salvia miltiorrhiza* in ameliorating inflammatory induction during endometritis.

### 3.7 Evaluation of molecular targets associated with blood stasis

**FIGURE 7:**
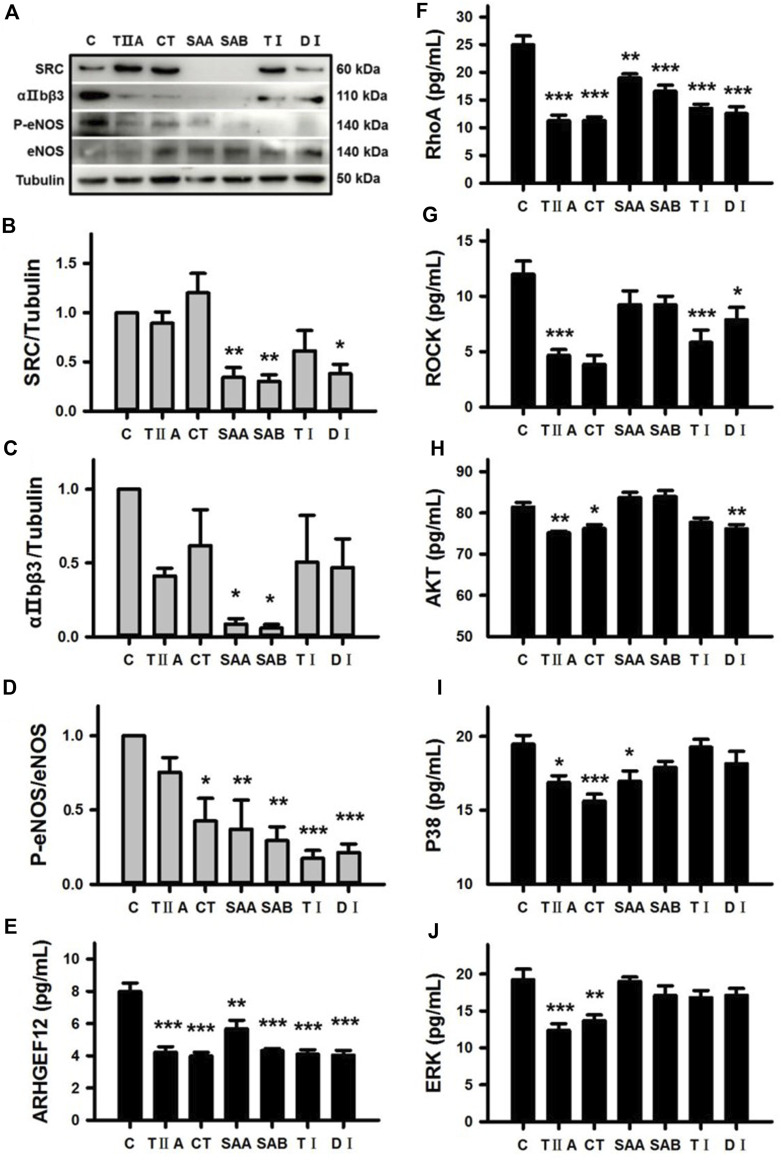
Verification of the effects of SMMCs on Platelet activation pathway targets. Western blotting assay of platelets from dairy cows that were cultured for 24 h in the presence or absence of six SMMCs. The platelet protein expression of SRC, αIIbβ3, P-eNOS and eNOS were evaluated, and tubulin was also tested as a loading control **(A)**. Quantification of the relevant ratios of SRC/Tubulin **(B)**, αIIbβ3/Tubulin **(C)** and P-eNOS/eNOS **(D)**. The effect of SMMCs on platelet protein concentration of ARHGEF12, RhoA, ROCK, AKT, P38 and ERK were evaluated by ELISA of cell lysates **(E–J)**. Values represent means ± SE (^*^ *p* < 0.05, ^**^ *p* < 0.01, ^***^ *p* < 0.001 vs. control group; *n* = 3 for B-D; *n* = 5 for **(E–J)**.

To confirm the predicted functions of all six SMMCs in modulating the Platelet activation pathway (Supplementary Figure S2), we performed Western blotting of SRC, αIIbβ3 and p-eNOS/eNOS expression in platelets from dairy cows. The results demonstrate that SAA, SAB and DI downregulated SRC expression; SAA and SAB downregulated αIIbβ3 expression; and five of the six SMMCs (CT, SAA, SAB, TI, and DI) inhibited eNOS phosphorylation (Figures 7A–D). Furthermore, all of the SMMCs downregulated platelet protein concentrations of ARHGEF12 and RhoA (Figures 7E,F), while different subsets of the SMMCs downregulated ROCK, AKT, P38 and ERK, as assessed by ELISA (Figures 7G–J). The effect of the SMMCs on PTGS1 protein expression could not be confirmed (data not shown). However, the results verify the roles of several SMMCs in regulating the expression of platelet signaling pathway components, thus supporting the role of *Salvia miltiorrhiza* in preventing blood stasis.

### 3.8 Evaluation of molecular targets associated with energy

**FIGURE 8:**
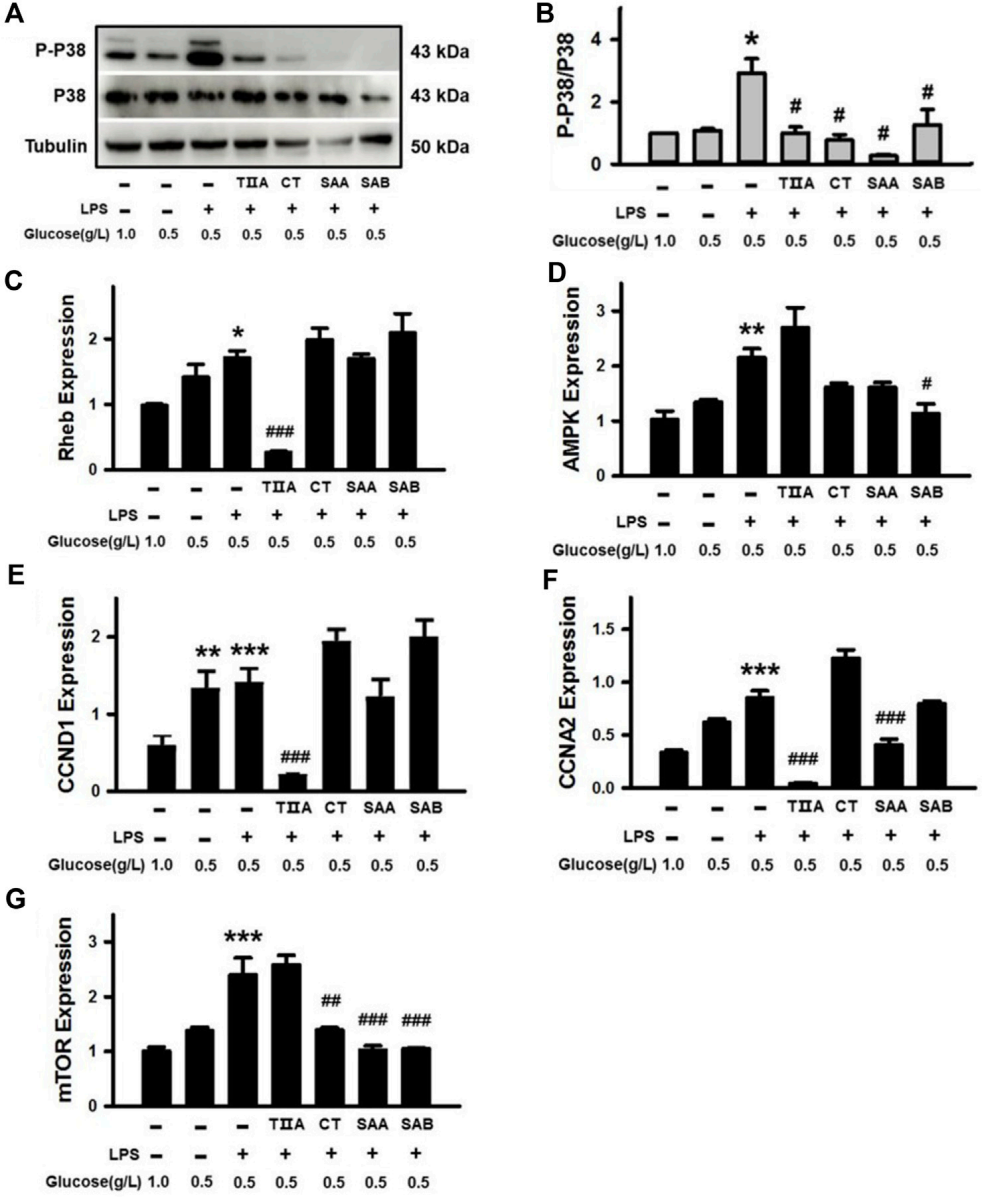
Verification of the effects of SMMCs on Thermogenesis and AMPK signaling pathway targets. Western blotting assay of BEECs cultured for 24 h with or without 100 μg/mL LPS and four SMMCs in normal DMEM (1.0 g/L) or low glucose DMEM (0.5 g/L). The protein phosphorylation of P-P38/P38 were evaluated, and tubulin was also tested as a loading control **(A)**. Quantification of the relevant ratio of P-P38/P38 **(B)**. The relative gene expression of Rheb, AMPK, CCND1, CCNA2 and mTOR was determined by qRT-PCR **(C–G)**. Values represent means ± SE (^*^ *p* < 0.05, ^**^ *p* < 0.01, ^***^ *p* < 0.001 vs. control group; ^#^ *p* < 0.05, ^##^ *p* < 0.01, ^###^ *p* < 0.001 vs. LPS+0.5 g/L glucose group; *n* = 3).

Given the central role of P38 in the Thermogenesis signaling pathway (Supplementary Figure S3), which is associated with energy balance, we evaluated the effect of TIIA, CT, SAA and SAB on P38 activation in BEECs under NEB conditions. The results demonstrate that LPS-triggered P38 phosphorylation was inhibited by all four SMMCs in cells cultured in low glucose media (Figures 8A,B). We further evaluated effects on the mRNA expression of Rheb, a mediator of the mTOR signaling pathway that is a predicted SMMC target in both the Thermogenesis and AMPK signaling pathways; as well as AMPK, CCND1, and CCNA2, which are predicted SMMC targets in the AMPK signaling pathway (Supplementary Figures S3, S4). The results demonstrate that TIIA reversed the increased expression of Rheb and CCND1; SAB reversed the increased expression of AMPK; and both TIIA and SAA reversed the increased expression of CCNA2 in cells stimulated with LPS under NEB conditions (Figures 8C–F). Additionally, CT, SAA, and SAB reversed the LPS and NEB-mediated increase in mTOR expression (Figure 8G), while all SMMCs failed to affect PCK1, PPARG and FGFR1 gene expression (data not shown). The above results indicate that SMMCs can restore cell energy balance by suppressing P38, Rheb, Cyclin D1 and Cyclin A, which may be coupled with the suppression of AMPK and mTOR signaling.

## 4 Discussion

Based on the “holistic concept” of TCVM, the main etiologies for endometritis are Qi deficiency (NEB), blood stasis, and external evil invasion. In accordance with TCVM theory, the uterine inflammation that endometritis dairy cows suffer from is typically caused by bacterial infection, a form of external evil invasion, and is usually accompanied by energy deficiency and blood stasis (Dervishi et al., 2021; Lean et al., 2023). To validate the connection of endometritis with NEB and blood stasis, we performed experiments both on BEECs under NEB condition and on rats with blood stasis. The results indicate that energy deficiency and blood stasis each can trigger inflammatory factor release, thus verifying the association between Qi deficiency, blood stasis, and external evil invasion. These findings support the use of a holistic therapeutic strategy that can simultaneously improve all three disease processes in endometritis.

We observed that after perfusion with *Salvia miltiorrhiza*, the uterine inflammation, blood stasis and NEB of endometritis dairy cows were all relieved, though the endometritis was not completely cured by treatment with *Salvia miltiorrhiza*. This may be explained in part by the limited dosage and 3-day intervention that was used. According to our previous study, endometritis can be completely cured with a complicated prescription that contains *Salvia miltiorrhiza* (Li et al., 2019), suggesting that its effect may also be improved by co-treatment with other herbal remedies. Furthermore, due to the comprehensive composition of *Salvia miltiorrhiza*, it is difficult to clarify its molecular components that contribute to endometritis therapy. As a traditional Chinese medicine for promoting blood circulation and removing blood stasis, components of *Salvia miltiorrhiza* that promote blood circulation, regulate energy and inhibit inflammation have been reported (Lin et al., 2013), including TIIA, CT, DI, SAA, SAB and TI (Feng et al., 2021). Therefore, we employed the above six SMMCs in the present study to understand factors that provide therapeutic value to *Salvia miltiorrhiza* in endometritis treatment. However, future studies to evaluate the efficacy of combinations of SMMCs at varying doses and with extended treatment regimens may help to optimize the treatment of endometritis in dairy cows.

The process of delivery consumes a large amount of energy and causes Qi deficiency (Bao et al., 2021). For decades, endometritis was considered to only relate to microorganism infection (Sheldon et al., 2018). However, increasing numbers of studies have demonstrated that the body’s energy status contributes to *postpartum* endometritis infection. With in-depth investigation, the correlation between inflammation and energy has been further clarified (Hussain et al., 2023). According to the present study, treatment of BEEC with LPS causes energy loss as evidenced by decreased ATP levels, and this energy loss is intensified by culture in low glucose media, which replicates the state of NEB during endometritis. Furthermore, we demonstrated that under NEB conditions, BEECs release higher levels of inflammatory factors both spontaneously, and in response to LPS treatment. These results further confirm the crosstalk between energy and inflammation in BEECs. Moreover, four of the SMMCs (TIIA, CT, SAA, and SAB) reversed the effect of LPS on ATP and/or inflammatory factors under NEB conditions, thus providing a mechanistic basis for the activity of *Salvia miltiorrhiza.*

Based on intensive studies investigating blood stasis, inflammatory factors in the blood circulation play important roles in the tissue inflammation process (Marshall, 2001; Levi et al., 2004). Inflammatory factors released from platelets, erythrocytes and leukocytes can indirectly cause tissue damage, such as edema and exudation (Di Virgilio et al., 2001; Siddall et al., 2017; Iba and Levy, 2018). Consistent with the former studies, we observed that uterine inflammatory factors in the rat blood stasis model were obviously higher than in control rats. The results suggest that even in absent of bacterial infection, blood stasis can induce uterine inflammation. Moreover, all six of the SMMCs were effective at inhibiting aggregation of platelets, thus supporting the role for *Salvia miltiorrhiza* in preventing blood stasis.

**FIGURE 9:**
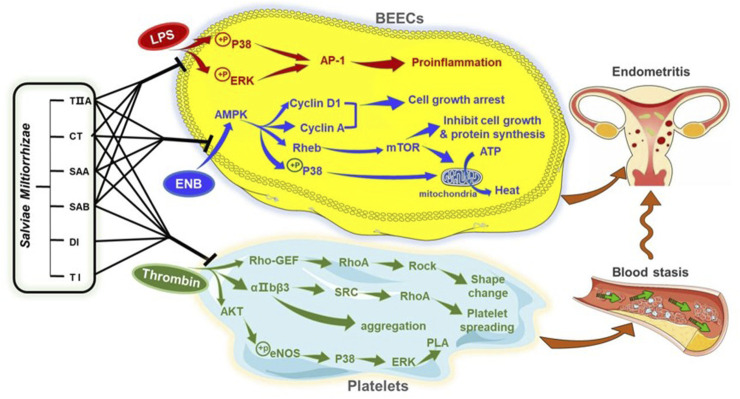
Proposed mechanism of *Salvia miltiorrhiza* in endometritis therapy. The results of this study indicate that SMMCs target three main processes that contribute to endometritis in dairy cows: Inflammation, as modeled by LPS exposure; NEB, as modeled by glucose deprivation; and blood stasis, as modeled by thrombin-triggered platelet aggregation. Molecular mediators that may be targeted by *Salvia miltiorrhiza* are shown. These three processes may serve as a biomedical explanation for the TCVM characteristics of Qi deficiency, blood stasis, and external evil invasion.

To provide additional insight into the mechanisms of the SMMCs, we predicted molecular targets by network pharmacological analysis. The putative targets for the SMMCs were centered in four pathways that support the principles of TCVM: the Toll-like receptor signaling pathway relates to inflammation; the Platelet activation pathway relates to blood stasis; and the AMPK signaling and Thermogenesis pathways relate to energy. We confirmed the effects of various SMMCs in modulating the expression or activity of several predicted targets within each of these pathways, which supports a model in which *Salvia miltiorrhiza* co-regulates Qi deficiency, blood stasis, and external evil invasion (Figure 9; Supplementary Figures S1–S4).

According to our experimental results, TIIA, CT, SAA, and SAB inhibit the phosphorylation of P38 and ERK and subsequent upregulation of the AP-1 subunits FOS and JUN, each of which are key molecules in the Toll-like receptor signaling pathway in LPS-induced BEECs. The ability of the 4 SMMCs to inhibit these signaling molecules may help to explain the anti-inflammatory effects of *Salvia miltiorrhiza*. Notably, P38 is also a key signaling molecule within the Platelet activation and Thermogenesis pathways. Consistently, our results demonstrated that P38 phosphorylation was also reduced by the above 4 SMMCs under NEB conditions, and that P38 expression was suppressed by TIIA, CT, and SAA in cultured platelets. These results support the crosstalk between inflammation, energy deficiency and blood stasis and suggest that P38 may comprise a key molecular mediator in the therapeutic effect of *Salvia miltiorrhiza*.

Notably, our results also demonstrated that all six of the SMMCs are effective in inhibiting thrombin-induced platelet aggregation. The antiplatelet effect has been shown to be achieved by preventing platelet shape change, spreading and aggregation triggered through Rho-GEF-RhoA-Rock, αIIbβ3-SRC-RhoA, and AKT-eNOS-P38-ERK signaling pathways (Aburima et al., 2013), and our results show that several molecules within these pathways are targeted in platelets by two or more SMMCs. In contrast, the SMMCs failed to influence *in vitro* plasma coagulation. This may be due to our *in vitro* assessment system, which lacked the complexity of *in vivo* studies. Coagulation factors in blood are mostly released from blood cells such as platelets, erythrocytes and leukocytes (George, 2008). Therefore, the *in vivo* activity of *Salvia miltiorrhiza* in prolonging the clotting time may be attributed to coagulators released from a variety of blood cells. Previously studies have demonstrated that AKT-eNOS-P38-ERK signaling does not directly regulate platelet function but activates cytosolic phospholipase A2 (PLA) (Chen et al., 2009). PLA is a rate-limiting enzyme of lipid mediators such as arachidonic acid, prostaglandin and platelet activating factor, as well as inflammatory mediators (Liu and Chang, 2009). Thus, it would be worth evaluating whether crosstalk between blood stasis and inflammation mediated by AKT-eNOS-P38-ERK-PLA may be restored by *Salvia miltiorrhiza*.

In addition to their role in counteracting NEB via P38, we also examined the effect of TIIA, CT, SAA and SAB in regulating energy balance via a comprehensive assessment of molecules within the AMPK signaling pathway, which is known to promote metabolic adaptation upon energy stress (Zhang et al., 2023). In NEB conditions, activation of AMPK triggers a series of signaling events: Cyclin D1 and Cyclin A increase induced cell growth arrest; Rheb and mTOR induce inhibition of cell growth and protein synthesis; and P38 phosphorylation and mTOR increase induce mitochondrial conversion of ATP into heat (Turchi et al., 2020). Each of these signaling mediators in NEB conditions were restored by one or more of the SMMCs, thus validating multiple potential mechanisms of SMMCs in regulating the energy balance in endometritis.

In conclusion, *postpartum* endometritis in dairy cows is not simply induced by bacterial infection. *Postartum* energy deficiency and blood stasis also contribute to the generation of inflammation. *Salvia miltiorrhiza* may be beneficial in the treatment of endometritis and exert its effects through anti-inflammation, energy re-balance, and blood circulation. P38 and AKT-eNOS-P38-ERK-PLA may be key molecular signals that mediate the crosstalk of inflammation with blood stasis and energy deficiency.

## Data Availability

The original contributions presented in the study are included in the article/Supplementary Material, further inquiries can be directed to the corresponding authors.

## Abbreviations

SMMCs, *Salvia miltiorrhiza* main components; TCVM, traditional Chinese veterinary medicine; NEB, negative energy balance; PMN, polymorphonuclear neutrophil; VDS, vaginal discharge score; APTT, active part thrombin time; PT, prothrombin time; TT, thrombin time; FIB, fibrinogen; BEECs, Bovine endometrial epithelium cells; TIIA, tanshinone IIA; CT, cryptotanshinone; DI, dihydrotanshinone I; SAA, salvianolic acid A; SAB, salvianolic acid B; TI, tanshinone I; WPs, washed platelets; LPS, lipopolysaccharide; PLA, phospholipase A2.
