# Supplementary figures and images for: Salvia miltiorrhiza ameliorates endometritis in dairy cows by relieving inflammation, energy deficiency and blood stasis
Source: Front Pharmacol. 2024 Apr 3;15:1349139. doi: 10.3389/fphar.2024.1349139 (PMC11021767; doi:10.3389/fphar.2024.1349139)

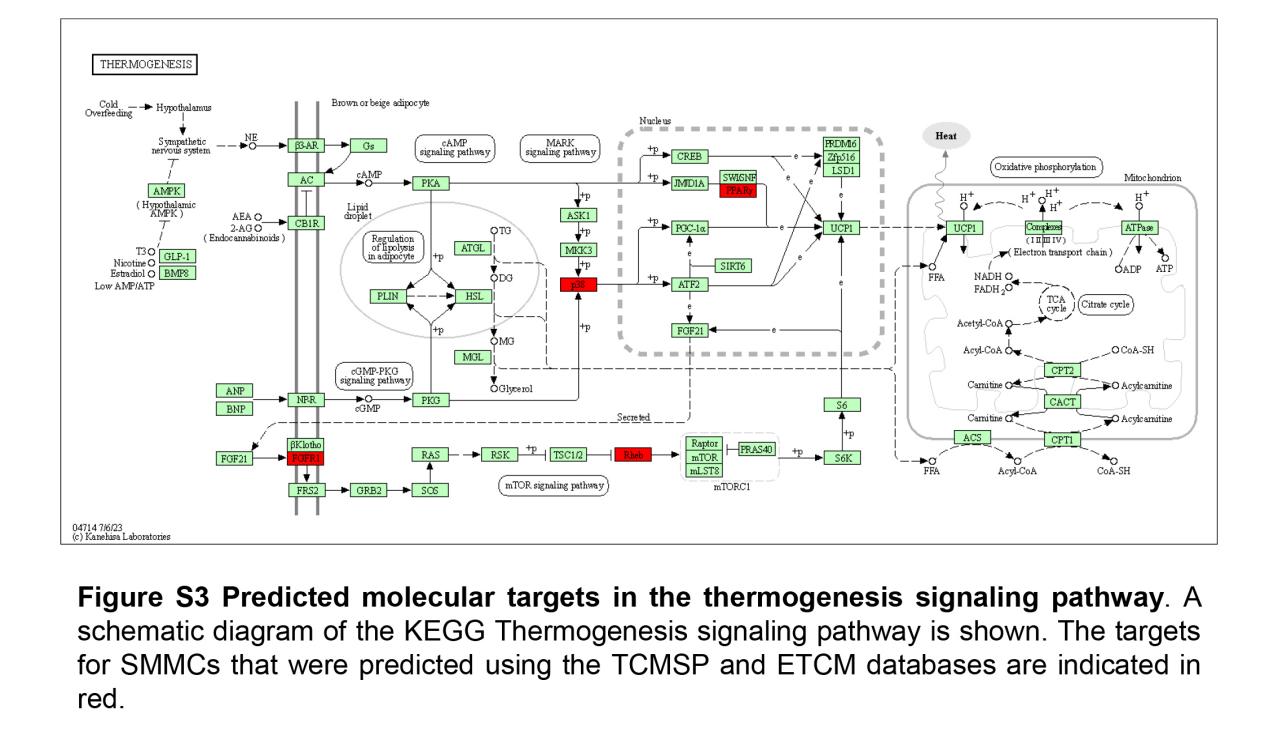

Supplement: Supplementary file 1 [file Image3.JPEG]

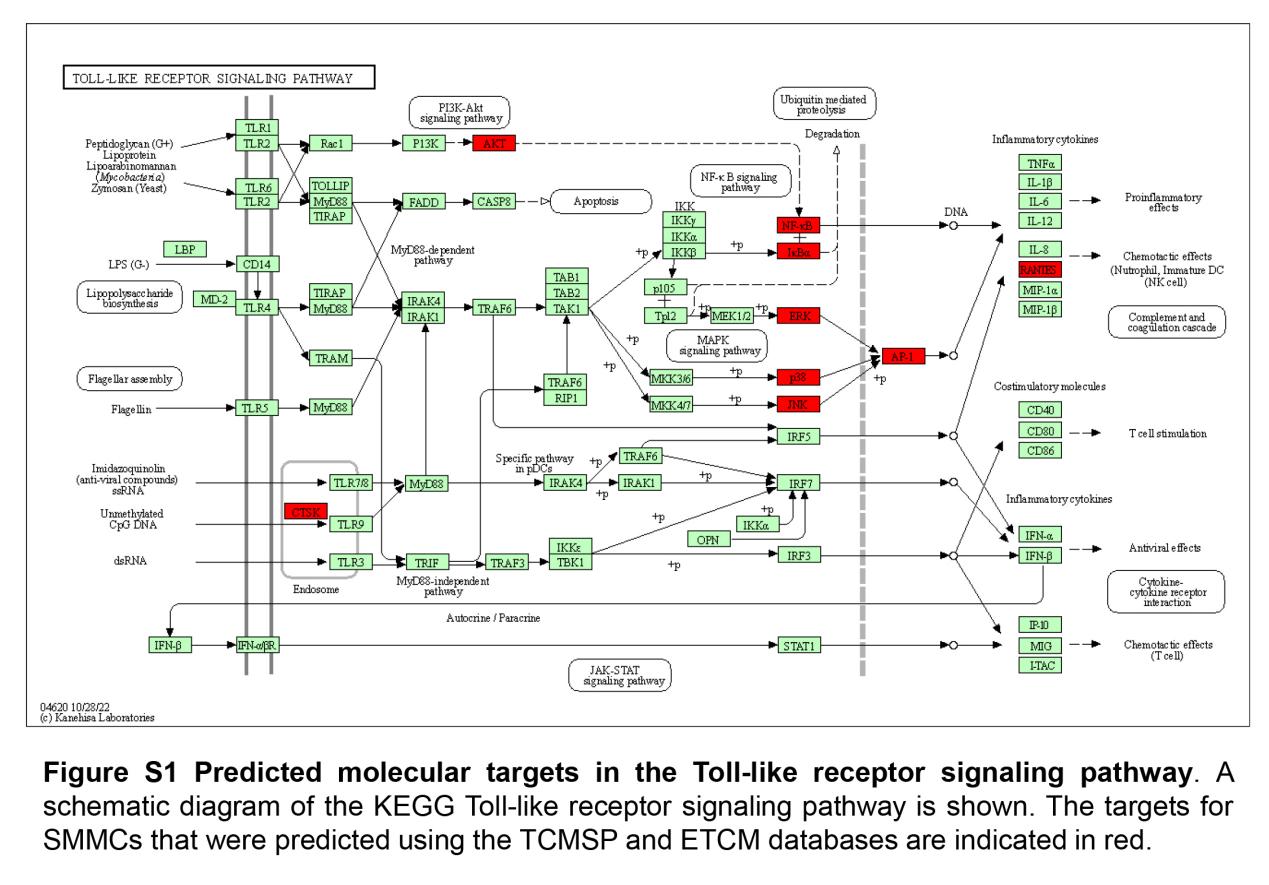

Supplement: Supplementary file 3 [file Image1.JPEG]

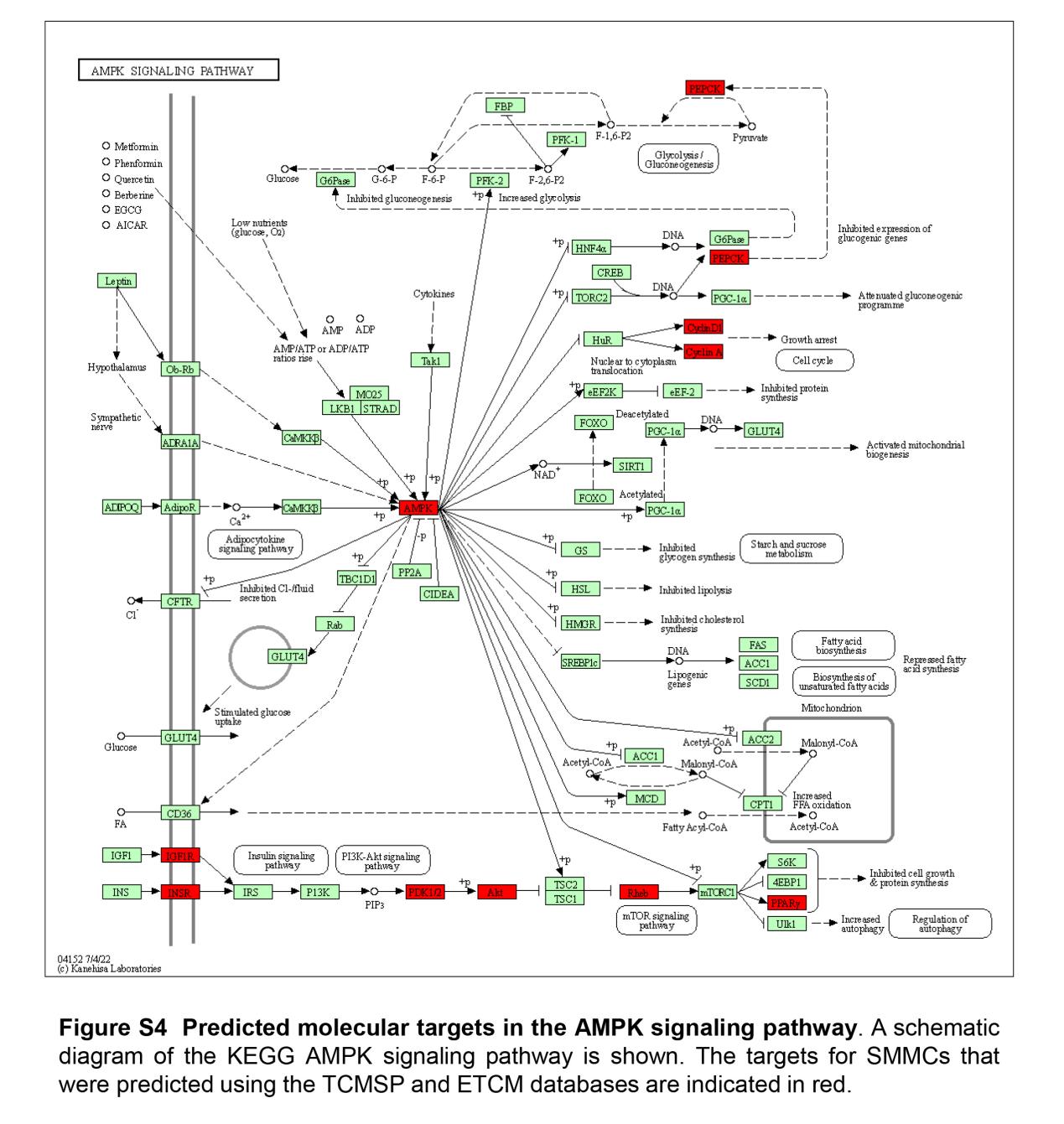

Supplement: Supplementary file 4 [file Image4.JPEG]

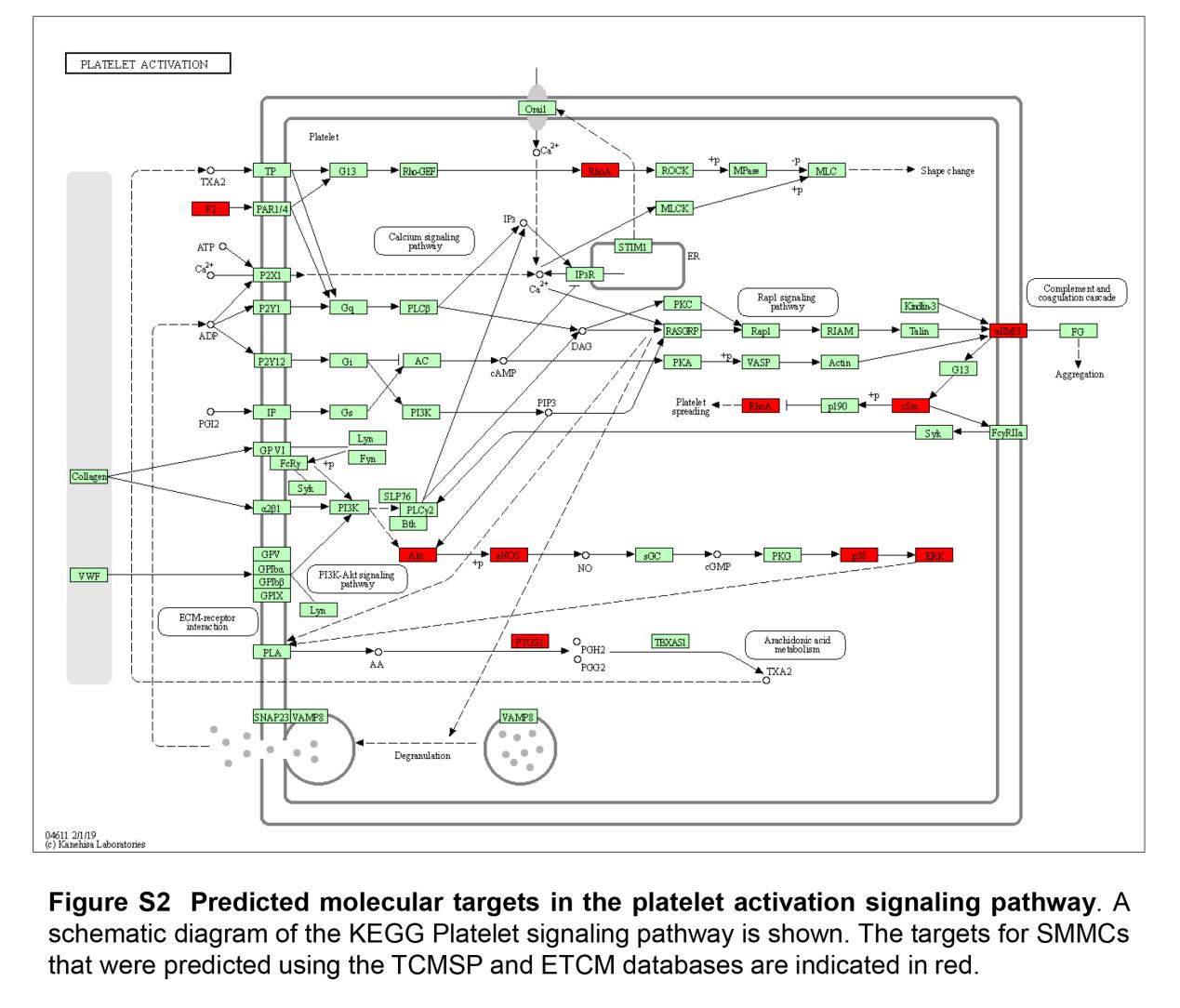

Supplement: Supplementary file 5 [file Image2.JPEG]
