# Supplementary material for: Salvia miltiorrhiza ameliorates endometritis in dairy cows by relieving inflammation, energy deficiency and blood stasis
Source: Front Pharmacol. 2024 Apr 3;15:1349139. doi: 10.3389/fphar.2024.1349139 (PMC11021767; doi:10.3389/fphar.2024.1349139)
Supplement: Supplementary file 2 [file Table1.DOCX]

**Table S1 Primer sequences for mRNAs**

| **Primers** | **Forward (5’–3’)** | **Reverse (5’–3’)** |
| --- | --- | --- |
| JUN | *TGCAAACGTTTTGAGGCGAG* | *GGGCTTTAGTCCTCGGACAC* |
| FOS | *AAAGGCGAATCCGAAGGGAA* | *TCTAGTTGGTCTGTCTCCGCT* |
| CTSK | *AGAGCTGACTTCCGCAATCC* | *GAGACCCCACATCCTGTTGA* |
| CCL5 | *CTCCATGGCAGCAGTTGTCTTT* | *AGCTGCTTAGGACAAGAGCG* |
| PPARg | *GACGACAGACAAATCACCGT* | *CAGGGGACTGATGTGCTTGA* |
| mTOR | *CGGCAACTTGACCATCCTCT* | *GAAGGCATCAATCTTGCGGG* |
| AMPK | *CGGCAAAGTGAAGGGGATGG* | *CCCTGTGGGCTATACAGCAA* |
| FGFR1 | *GCAGACAGGTAACAGTGTCG* | *ATATTCAGAGACCCCGGCCA* |
| RHEB | *CTGAGGAGGCCGCTAAGATG* | *AACGAGGATTTCCCCACAGAC* |
| CCND1 | *GCAGATCATCCGCAAACACG* | *GTTGGAAATGAACTTCACGTCTGT* |
| CCNA2 | *CGTACCTTAGGGAAATGGAGGTTA* | *CAGGGTCTCGTTCTGCAGTT* |
| PCK1 | *CCAACTCACGGTTCTGCACT* | *ACCAGAGGGACACCAACAGG* |
| 36b4 | *GGCAGCATCTACAACCCTGA* | *CAGATGCGACGGTTGGGTAA* |

**Table S2 *Salvia miltiorrhiza* active ingredient content**

| **Sample** | | **Retention time**  **(min)** | **Peak area** | **Concentration**  **(mg/mL)** | **Average concentration**  **(mg/mL)** |
| --- | --- | --- | --- | --- | --- |
| SAB | reference | 23.950 | 333927 | 0.06 |  |
|  | 1 | 23.751 | 3465644 | 62.27 | 62.50 |
|  | 2 | 23.705 | 3586865 | 64.45 |  |
|  | 3 | 23.828 | 3383387 | 60.80 |  |
| SAA | reference | 8.747 | 445883 | 0.06 |  |
|  | 1 | 8.724 | 314010 | 4.23 | 3.55 |
|  | 2 | 8.720 | 252048 | 3.40 |  |
|  | 3 | 8.726 | 225271 | 3.03 |  |
| TⅠ | reference | 17.882 | 243672 | 0.06 |  |
|  | 1 | 17.872 | 57032 | 1.40 | 1.40 |
|  | 2 | 17.864 | 58428 | 1.44 |  |
|  | 3 | 17.853 | 55664 | 1.37 |  |
| CT | reference | 9.162 | 1021962 | 0.18 |  |
|  | 1 | 9.159 | 92069 | 1.62 | 1.66 |
|  | 2 | 9.162 | 96969 | 1.71 |  |
|  | 3 | 9.162 | 94321 | 1.66 |  |
| TⅡA | reference | 45.335 | 2394975 | 0.18 |  |
|  | 1 | 45.287 | 2176356 | 16.36 | 16.10 |
|  | 2 | 45.291 | 2124843 | 15.97 |  |
|  | 3 | 45.323 | 2125644 | 15.96 |  |
| DⅠ | reference | 5.952 | 1279586 | 0.09 |  |
|  | 1 | 5.954 | 14650 | 0.10 | 0.08 |
|  | 2 | 5.953 | 10877 | 0.07 |  |
|  | 3 | 5.953 | 11678 | 0.08 |  |

**Table S3 Diagnostic criteria for dairy cows**

| **Group** | **Animal**  **number** | **Before treatment** | | **After treatment** | |
| --- | --- | --- | --- | --- | --- |
|  |  | **VDS** | **PMN (%)** | **VDS** | **PMN (%)** |
| Healthy | 1 | 0 | 3.10 | 0 | 3.14 |
|  | 2 | 0 | 3.08 | 0 | 4.16 |
|  | 3 | 0 | 2.42 | 0 | 3.75 |
|  | 4 | 0 | 2.91 | 0 | 2.91 |
|  | 5 | 0 | 3.71 | 0 | 3.81 |
|  | 6 | 0 | 4.76 | 0 | 4.68 |
|  | 7 | 0 | 3.71 | 0 | 4.92 |
|  | 8 | 0 | 4.54 | 0 | 3.61 |
|  | 9 | 0 | 4.27 | 0 | 3.94 |
|  | 10 | 0 | 3.84 | 0 | 4.35 |
| Endometritis | 1 | 2 | 9.71 | 3 | 10.51 |
|  | 2 | 2 | 8.64 | 2 | 13.74 |
|  | 3 | 2 | 9.74 | 2 | 8.41 |
|  | 4 | 2 | 10.24 | 2 | 8.41 |
|  | 5 | 3 | 7.69 | 3 | 6.71 |
|  | 6 | 2 | 8.46 | 2 | 7.64 |
|  | 7 | 2 | 9.32 | 3 | 12.48 |
|  | 8 | 2 | 10.43 | 3 | 12.74 |
|  | 9 | 3 | 9.27 | 2 | 10.52 |
|  | 10 | 2 | 8.45 | 2 | 7.64 |
| *Salvia miltiorrhiza* | 1 | 2 | 9.24 | 1 | 7.42 |
|  | 2 | 2 | 9.73 | 2 | 7.34 |
|  | 3 | 2 | 6.24 | 2 | 4.24 |
|  | 4 | 3 | 10.81 | 2 | 4.41 |
|  | 5 | 2 | 9.71 | 1 | 5.07 |
|  | 6 | 3 | 12.62 | 2 | 6.17 |
|  | 7 | 2 | 9.37 | 1 | 7.51 |
|  | 8 | 2 | 11.48 | 1 | 6.37 |
|  | 9 | 2 | 9.14 | 2 | 7.84 |
|  | 10 | 2 | 8.37 | 1 | 6.38 |

**Table S4 Network analyzed SMMC targets**

| **SMMC** | **Molecular ID** | **Targets** |
| --- | --- | --- |
| TⅡA | MOL007154 | ALB APOA2 STS MAPK1 MAPK10 MMP3 CA2 BMP2 BCHE ENSG00000244255 PPIA CASP7 MAPK8 KIF1 AKR1C2 MAPKAPK2 TTR ESR1 CCL24 PDPK1 AR GC CDK5R1 METAP2 RXRA RORA PDE4D PGR EGFR MAOB PDE4B SRC CCNA2 BCAT2 ESRRG MAPK14 HSP90AA1 PNMT ADAM17 DPP4 TYMS SHBG TGFBR1 DHODH F2 PPARD MMP13 AKR1B DUSP6 CHEK1 SULT2A1 SEC14L2 PTPN11 ESR2 PPARG HSD11B1 HPGDS GSK3B GSTP1 HSD17B1 PPP5C NR3C2 GSTA1 AKR1C1 LTA4H CDK2 CES1 WAS FNTA F10 NR1H4 ADH1C HDAC8 SOD2 PKI CDK6 MDM2 CALM2 GSR PTPN1 ADH5 EPHX2 SULT2B1 CYP2C9 MMP8 MET THRB BACE1 TTPA PGF CTNNA1 ADK NR1H2 MIF MTAP RBP4 EPHB4 TRAPPC3L DAPK1 FABP6 SORD CCNT1 KDR JAK3 ITK NOS3 FKBP1A ESRRA ERBB4 PPARA HMGCR FECH NQO1 SULT1E1 AKR1C3 SERPINA1 ABO DRD1 CHRM3 CHRM1 SCN5A CHRM5 PTGS2 CHRM4 OPRD1 ACHE ADRA1D CHRM2 ADRB2 OPRM1 CHRNA7 NCOA1 RELA BCL2 FOS CDKN1A MMP9 JUN AHSA1 CASP3 TP53 NFKBIA FASN EDNRA EDN1 CYP3A4 MYC CYP1A1 NR1I2 NPM1 ECE1 PARP4 CALCR ITGB3 |
| CT | MOL007088 | BMP2 MAPK1 STS ALB AKR1C2 APOA2 CES1 PPIA CA2 PDPK1 CCL24 EPHB4 MAPK10 ENSG00000244255 ESR1 CDK5R1 F2 AR AKR1B1 ESRRG MAPK14 ADAM17 CCNA2 CDK2 KIF11 FGFR1 MAPK8 MIF SHBG PPARG HSP90AA1 PNMT TTR SRC PDE4D TYMS MMP8 MAOB GC CTSB SEC14L2 PPP5C F10 PGR EGFR MMP13 PTPN1 ANXA5 ESR2 NOS3 BACE1 CHEK1 RORA TGFBR1 PTPN11 SULT2A1 CASP3 TNNC1 PDE4B HSD11B1 GSR HSD17B1 PCK1 PPARD NR3C2 WAS CFD DPP4 DUSP6 RXRA NR1H2 HPGDS METAP2 AKR1C1 PKIA RARA HMGCR GSK3B PGF NR1H3 LSS HDAC8 FGFR2 SULT1E1 CALM2 EPHX2 MET NR1I3 KDR CTSK NR1H4 SOD2 TRAPPC3L ADH5 CTNNA1 RBP4 DAPK1 DHODH ADK MMP12 CDK6 PDE3B GSTP1 MDM2 THRB TTPA CDA ADH1C SYK REN MTAP AKR1C3 CCNT1 MMP7 CYP2C9 BCHE PTGS1 DRD1 CHRM3 CHRM1 SCN5A CHRM5 PTGS2 CHRM4 OPRD1 ADRA1D CHRM2 ADRA1B ADRB2 TOP2A OPRM1 CHRNA7 NCOA2 NCOA1 RELA STAT3 CCND1 BCL2L1 CD40LG APP EDN1 BIRC5 |
| SAA | MOL007136 | APOA2 STS ALB ENSG00000244255 MAPK14 PPIA ESR1 FAP TTR BCHE CCL24 SNRPA CA2 EPHB4 GSTP1 MAPK10 MAPK8 NR1H2 CDK2 APCS CHEK1 EGFR PGR SPARC NPR3 HSD17B11 GBA CA1 DDX6 MMP3 AKR1B1 AKR1C3 CALM2 CMA1 AKR1C2 PNP CASP3 ANXA5 AURKA SELP ESR2 SRC PPARG CTSD MAPK1 PDE5A CES1 PDE4B CDK5R1 MAPKAPK2 BMP2 CTSS F2 BACE1 NOS3 HSP90AA1 KIF11 CYP19A1 NQO2 METAP2 MAOB HSD17B1 CHIT1 DAPK1 MIF CTSV CSNK1G2 QPCT PLAU TREM1 CCNA2 SHBG PLK1 MMP13 IMPDH2 HSD11B1 CA12 ISG20 RXRA GC LTA4H PKIA ADH1C GSR TGFBR1 PTPN1 FGFR1 PNPO MTAP ADH1B PNMT LCN2 FABP4 ESRRG ANG AMD1 BCAT2 PDPK1 F10 PPP5C PDE4D PAH KDR AR SEC14L2 ICAM2 LTF RORA ADAM17 DHODH TYMS METAP1 NQO1 CFD ADH5 PPARD CTSB CCBL1 GATM CASP7 NMNAT3 HSPA8 DHFR SOD2 AMY1B TYMP REN LPA BMP7 KIF5C MMP12 FNTA ME2 PLG LGALS7B GSK3B FHIT EEA1 CDK6 NR3C2 C1R MTHFD1 TGFBR2 ADK ARF1 MMP8 DPP4 ACE LSS SULT2A1 AZGP1 KIT PARP1 GLO1 IGF1R TNNC1 SORD SYK AHCY RAC2 AGXT PLA2G10 AKT1 DCK GALE PCK1 HPGDS MMP7 HMGCR CSNK2A1 PYGL PGF ELANE MET DUSP6 CTNNA1 PPARA B3GAT3 GALK1 ITK FGFR2 PLA2G2A PTPN11 HSPA1B HDAC8 CLK1 FABP5 FABP6 BST1 CDA HK1 AMY2A JAK3 CTSK LDHB ABO CPB1 CBR1 TNK2 F7 ZAP70 PDE3B ACPP MDM2 EPHX2 ALDH2 NR1H4 FGF1 MAN1B1 CTSG NR1I3 RNASEL TTPA ATOX1 RHEB RHOA BLVRB IMPA2 SERPINA1 REG1A RBP4 XIAP WAS THRB HPN CCNT1 AKR1C1 CCL5 FABP3 IL2 HADH FABP7 CSK GSTA1 MMP9 PPCDC RARA GLRX DTYMK UCK2 OTC EPHA2 APRT SETD7 TGM3 FKBP1A SULT1E1 HCK SRR PSPH PTK2 S100A9 ACE2 CBSL PDHB PRKCQ INSR SULT2B1 NMNAT1 RAB11A IMPDH1 ARHGAP1 C8G CANT1 PRSS1 CCND1 BCL2 CDKN1A EIF6 COL7A1 PYGM |
| SAB | MOL007074 | TTR MIF MMP3 CA2 AKR1B1 GBA GSTP1 SRC BCHE MAPK14 FABP4 PDE4B EPHB4 APCS ESR1 MAPK10 CTSD SNRPA F2 B3GAT3 CA1 BCAT2 PPIA PNP NR1H2 CHEK1 PTPN1 ALB TYMS MMP13 CCBL1 AURKA ARF1 CES1 SELP CALM2 PSPH ESR2 MAPK8 SPARC IMPDH2 NPR3 FAP EGFR RTN4R LPA KIF5C ME2 LCN2 TGFBR2 ANG CMA1 PLA2G2A ICAM2 CCL24 KIF11 TREM1 CTSV PLG ADAM17 DDX6 ANXA5 F10 ENSG00000244255 CTSS CDK2 MAOB PNPO CHIT1 CTSB AR CYP19A1 METAP2 MAPKAPK2 CCNA2 PGR MAPK1 GSR F7 RNASEL BACE1 CSNK2A1 HSPA8 FABP5 CPB1 HMGCR DPP4 HSD17B1 PLAU DHFR IGF1R AMY1B CA12 NR3C2 ESRRG KDR SORD NQO1 CASP7 SHBG GC LTF PAH PDE4D RNASE3 CSNK1G2 HSP90AA1 PPP5C BMP7 AZGP1 PCK1 AMD1 CFD AMY2A FGFR1 TNNC1 EEA1 NOS3 HSD11B1 TGFBR1 C1R FGF1 DHODH ADH1B CRAT PLK1 MTAP TYMP SOD2 OTC CDK5R1 SULT2A1 STS C8G LGALS7B MTHFD1 RORA PDE5A DCK ADH5 GSK3B LSS PPARD APOA2 FHIT PPARG ACE2 GLRX NQO2 CTSK FNTA ACPP FABP3 AKT1 ISG20 C1S TGM3 PARP1 CDK6 PDPK1 ALDOA PAK6 REN GSTT2B HK1 IMPA2 PYGL TPI1 MMP12 EPHX2 GALE DAPK1 FABP7 AKR1C1 EPHA2 PDHB CTNNA1 MET RXRA PGF DTYMK CBR1 LDHB AGXT ARSA PKIA RAB11A BST1 PTK2 PDE3B KIT AKR1C3 ARF4 MDM2 ABO CTSG REG1A PLEKHA4 AHCY ADK DPEP1 SHMT1 WAS RAC2 MMP8 HPN ADH1C MME TNK2 SERPINA1 CDA HPGDS MAN1B1 INSR FGFR2 PTPN11 SEC14L2 CASP3 SETD7 THRB UMPS UCK2 SYK MMP7 PNMT HSPA1B CYP2C9 ATOX1 ELANE GART NCS1 CLK1 SRR CDC42 ITK NMNAT1 RHOA MAPK12 HDAC8 ALDH2 ALAD GALK1 LYZ NR1H4 TTPA BPI HAGH FABP6 LGALS3 CBSL IMPDH1 PADI4 NR1I2 PPCDC SULT1E1 CYP2C8 CASP1 RHEB GSTO1 JAK3 MMP2 FKBP1A HPRT1 PPARA CAT GPI NR1H3 THRA RNASE2 APRT LTA4H GSTA3 JAK2 IL2 TGM2 CANT1 SULT2B1 ARHGAP1 |
| TⅠ | MOL007101 | APOA2 BMP2 CA2 ALB ESR1 STS AKR1C2 MMP3 PDPK1 F2 CDK5R1 MAOB AKR1B1 PPIA MAPK10 MAPK8 ENSG00000244255 CCL24 ESRRG CYP19A1 CCNA2 PPARD KIF11 PDE4D AR MIF PNMT SHBG ADAM17 PPARG EGFR ANXA5 SEC14L2 CDK2 PPP5C DUSP6 HSP90AA1 GC RORA TYMS PGR MAPK14 NOS3 MMP13 F10 SULT2A1 CHEK1 BCHE SRC PTPN11 FNTA TNNC1 TTR PDE4B ESR2 HSD11B1 MAPK1 ANG TGFBR1 CTSB WAS PCK1 NR3C2 GSR FGFR1 CFD DPP4 PDE3B METAP2 TRAPPC3L EPHX2 IGF1R AKR1C1 GSK3B PKIA RXRA PGF HDAC8 CES1 BACE1 PLAU CTNNA1 MMP8 TTPA HSD17B1 EPHB4 FGFR2 DHODH NR1H4 SYK ADK KDR HPGDS SORD THRB PTPN1 CTSK ITK AKR1C3 RBP4 REN PDE5A MET ADH1C CDA CYP2C9 CCNT1 CDK6 ADH5 MMP12 ERBB4 PLA2G10 CYP2C8 DRD1 CHRM3 CHRM1 SCN5A CHRM5 PTGS2 CHRM4 OPRD1 ACHE ADRA1D CHRM2 ADRB2 OPRM1 CHRNA7 NCOA1 RELA BCL2 FOS CDKN1A MMP9 JUN AHSA1 CASP3 TP53 NFKBIA FASN EDNRA EDN1 CYP3A4 MYC CYP1A1 NR1I2 NPM1 ECE1 PARP4 CALCR ITGB3 |
| DⅠ | MOL007157 | SERPIND1 AR PTGS2 RXRA DPP4 HSP90AA1 PRSS1 NCOA1 VEGFA ICAM1 VCAM1 CCL24 RUFY3 EIF2AK2 APIP F11 MTOR MDM2 EF1A2 DHPS MEX3D RAC1 AMD1 AHCY HK1 RNF31 PTPRN RPS3 SRP19 OBP2A S100A8 ACBD7 GSTA1 CALML3 CDCA8 ANP32B MEF2A PFN1 RAB5C EGF HDLBP XRN2 F2 DAB1 OXSM SNX27 CREBBP PPA1 UNG SIN3B TOX DDX58 BRD7 NR1D2 RGS18 ACTR3C CYB5B RUNX1T1 ALDOA PTPRJ SDHA GATB WARS2 EIF4E UPRT VAV1 HUS1 AK6 GOT1 DAPK3 GPHN CALM2 CSDE1 GSTO1 ZKSCAN5 RANBP2 UCN2 C4BPB ADAM17 EIF2B2 RUVBL1 TJP1 CAPN2 GRASP TPK1 REPS2 QPRT HSD11B1 TSEN54 PDPK1 OAZ1 MAOB TNNC2 MYNN HSD17B11 GALM SMOX OGG1 ALOX12 SYNCRIP FLOT2 IYD PTBP1 CES2 GSR ARHGAP11A XPOT PPP1R8 LDHAL6A F13A1 CCND1 ZPR1 DRAP1 UGDH P4HB ALK ARHGEF12 AGL PEBP1 NMT1 PRPS1 USP19 LGALS2 EDC3 HFE NGLY1 MKNK1 GCM1 TIMM9 SOD2 PRKD2 VPS4B TOP1 ROCK2 HMGB2 POLD4 NHP2L1 RGS6 ATIC S100P S100A11 GMPR2 SPA17 HNRNPLL RAB7A HARS2 PLA2G2A H3F3B TRPV6 CANT1 |

**Table S5 SMMC targets topological parameters**

| **Target** | **Betweenness** | **Closeness** | **Degree** |
| --- | --- | --- | --- |
| ACE | 1654.098306 | 0.001049318 | 57 |
| MMP2 | 1132.351318 | 0.001074114 | 77 |
| CTSD | 1427.987325 | 0.00101626 | 47 |
| CAT | 7242.428255 | 0.001129944 | 93 |
| KDR | 1099.508198 | 0.001036269 | 69 |
| EGF | 3152.46685 | 0.001138952 | 110 |
| TP53 | 15238.20663 | 0.001248439 | 160 |
| REN | 1138.976055 | 0.001001001 | 48 |
| EGFR | 9095.097375 | 0.001210654 | 140 |
| APP | 5387.964073 | 0.001084599 | 78 |
| AKR1B1 | 1654.480316 | 0.001014199 | 39 |
| PPARG | 2970.114542 | 0.001106195 | 88 |
| MTOR | 2266.685301 | 0.001109878 | 86 |
| CASP3 | 4445.132261 | 0.001166861 | 114 |
| JUN | 3665.442771 | 0.001164144 | 124 |
| FOS | 3242.017312 | 0.001070664 | 78 |
| ESR1 | 4648.295909 | 0.001136364 | 111 |
| PPARA | 2558.115351 | 0.001061571 | 67 |
| CYP3A4 | 2503.852651 | 0.001034126 | 55 |
| LCN2 | 1087.019212 | 9.22E-04 | 30 |
| AKT1 | 12912.91698 | 0.001277139 | 168 |
| PTGS2 | 1517.703623 | 0.001078749 | 82 |
| VCAM1 | 1238.02135 | 0.001019368 | 58 |
| F2 | 1334.672159 | 9.98E-04 | 48 |
| MMP9 | 3192.254468 | 0.001127396 | 106 |
| PLG | 1900.197379 | 0.001055966 | 63 |
| VEGFA | 3157.607416 | 0.001176471 | 124 |
| DPP4 | 1108.652168 | 9.64E-04 | 28 |
| NOS3 | 2163.536303 | 0.001081081 | 70 |
| EDN1 | 1452.205326 | 0.001030928 | 56 |
| ALB | 21361.44524 | 0.001282051 | 176 |
| MME | 879.2282876 | 9.43E-04 | 29 |
| RHOA | 3356.156287 | 0.001129944 | 97 |
| GSR | 1039.950389 | 0.001009082 | 38 |
| HPGDS | 4079.98449 | 0.001101322 | 84 |
| PNP | 1499.294363 | 9.20E-04 | 29 |
| CDC42 | 2218.756775 | 0.001088139 | 79 |
| WAS | 1639.94427 | 9.20E-04 | 30 |
| MAPK14 | 1342.731128 | 0.001067236 | 74 |
| STAT3 | 2082.892073 | 0.001132503 | 106 |
| P4HB | 983.5019985 | 9.94E-04 | 32 |
| SRC | 6111.918755 | 0.001177856 | 130 |
| AHCY | 1219.65134 | 9.26E-04 | 31 |
| LDHB | 801.0540924 | 9.35E-04 | 32 |
| GSTP1 | 1740.42649 | 0.00102145 | 46 |
| TPI1 | 2759.467228 | 0.001002004 | 50 |
| GART | 6033.235056 | 0.001007049 | 54 |
| DHFR | 2182.509109 | 0.001011122 | 38 |
| TYMS | 3262.648992 | 9.90E-04 | 46 |
| HPRT1 | 2085.186203 | 0.001012146 | 37 |
| JAK2 | 887.5468859 | 0.001039501 | 61 |
| MDM2 | 1180.321251 | 0.001076426 | 77 |
| HSP90AA1 | 11682.56818 | 0.00120048 | 134 |
| RPS3 | 1731.932652 | 9.49E-04 | 30 |
| HSPA8 | 1931.200414 | 0.001058201 | 58 |
| FASN | 1017.029983 | 9.80E-04 | 30 |
| SOD2 | 1396.948953 | 0.001038422 | 47 |
| CYP1A1 | 959.5167269 | 9.77E-04 | 42 |
| AR | 1297.364334 | 0.001037344 | 61 |
| CYP19A1 | 1344.515391 | 9.74E-04 | 36 |
| MAPK1 | 2323.101527 | 0.001104972 | 92 |
| NFKBIA | 1104.043271 | 0.001068376 | 71 |
| AURKA | 730.2688453 | 9.52E-04 | 33 |
| IL-2 | 902.6660022 | 0.001035197 | 55 |
| CCND1 | 1038.793493 | 0.001102536 | 86 |
| CREBBP | 1737.013988 | 0.001023541 | 60 |
| RAB7A | 1302.703889 | 9.87E-04 | 28 |
| CCNA2 | 1611.066636 | 0.001031992 | 54 |
| ANXA5 | 727.9750818 | 0.001078749 | 79 |
| PTPN11 | 1097.352172 | 0.001027749 | 57 |
| PTK2 | 792.1834171 | 0.001020408 | 58 |
| CTSB | 841.2110357 | 0.00101833 | 47 |
| TOP1 | 1077.021651 | 0.001005025 | 37 |
| PARP1 | 2591.616344 | 0.001040583 | 57 |
| BCL2 | 1078.616809 | 9.43E-04 | 27 |
| RELA | 1027.475404 | 0.001055966 | 66 |
| CHEK1 | 1188.717552 | 9.96E-04 | 43 |
| SERPINA1 | 2007.212953 | 9.90E-04 | 45 |
| RXRA | 772.5995327 | 9.66E-04 | 38 |
| EIF4E | 2680.583877 | 9.89E-04 | 41 |
| ARF1 | 1234.732564 | 9.40E-04 | 27 |
| CCL5 | 1270.298104 | 9.97E-04 | 48 |
| MYC | 5773.092545 | 0.001179245 | 124 |
| SDHA | 1198.163872 | 9.43E-04 | 26 |
| TGFBR1 | 1516.770539 | 9.60E-04 | 31 |
| GC | 955.684742 | 8.94E-04 | 27 |
| NCOA1 | 1602.684336 | 9.80E-04 | 43 |
| ITGB3 | 641.9360043 | 0.000988 | 49 |
| PTGS1 | 421.0813107 | 0.000923 | 21 |

# Table S6 Gene and protein names for the predicted SMMC targets in KEGG pathways

| **Gene name** | **Protein Name** |
| --- | --- |
| PCK1 | PEPCK |
| CCND1 | Cyclin D1 |
| CCNA2 | Cyclin A |
| MAPK12 MAPK14 | p38 |
| PPARG | PPARγ |
| FGFR1 | FGFR1 |
| AKT1 | AKT |
| RELA | NFκB |
| NFKBIA | IκB |
| MAPK1 | ERK |
| FOS JUN | AP-1 |
| MAPK8, MAPK10 | JNK |
| CTSK | CTSK |
| CCL5 | RANTES |
| ARHGEF12 | Rho-GEF |
| RHOA | RhoA |
| ROCK2 | ROCK |
| SYK | Syk |
| NOS3 | eNOS |
| PTGS1 | PTGS1 |
| SRC | cSrc |
| Rheb | Rheb |
| ITGB3 | αⅡbβ3 |
